# Pharmacological targeting of CSF1R inhibits microglial proliferation and prevents the progression of Alzheimer’s-like pathology

**DOI:** 10.1093/brain/awv379

**Published:** 2016-01-08

**Authors:** Adrian Olmos-Alonso, Sjoerd T. T. Schetters, Sarmi Sri, Katharine Askew, Renzo Mancuso, Mariana Vargas-Caballero, Christian Holscher, V. Hugh Perry, Diego Gomez-Nicola

**Affiliations:** ^1^ 1 Centre for Biological Sciences, University of Southampton, Southampton, UK; ^2^ 2 Institute for Life Sciences, University of Southampton, Southampton, UK; ^3^ 3 Division of Biomedical and Life Sciences, Faculty of Health and Medicine, Lancaster University, Lancaster, LA1 4YQ, UK

**Keywords:** Alzheimer’s disease, microglia, gliosis, neurodegeneration, inflammation

## Abstract

The proliferation and activation of microglial cells is a hallmark of several neurodegenerative conditions. This mechanism is regulated by the activation of the colony-stimulating factor 1 receptor (CSF1R), thus providing a target that may prevent the progression of conditions such as Alzheimer’s disease. However, the study of microglial proliferation in Alzheimer’s disease and validation of the efficacy of CSF1R-inhibiting strategies have not yet been reported. In this study we found increased proliferation of microglial cells in human Alzheimer’s disease, in line with an increased upregulation of the CSF1R-dependent pro-mitogenic cascade, correlating with disease severity. Using a transgenic model of Alzheimer’s-like pathology (APPswe, PSEN1dE9; APP/PS1 mice) we define a CSF1R-dependent progressive increase in microglial proliferation, in the proximity of amyloid-β plaques. Prolonged inhibition of CSF1R in APP/PS1 mice by an orally available tyrosine kinase inhibitor (GW2580) resulted in the blockade of microglial proliferation and the shifting of the microglial inflammatory profile to an anti-inflammatory phenotype. Pharmacological targeting of CSF1R in APP/PS1 mice resulted in an improved performance in memory and behavioural tasks and a prevention of synaptic degeneration, although these changes were not correlated with a change in the number of amyloid-β plaques. Our results provide the first proof of the efficacy of CSF1R inhibition in models of Alzheimer’s disease, and validate the application of a therapeutic strategy aimed at modifying CSF1R activation as a promising approach to tackle microglial activation and the progression of Alzheimer’s disease.

## Teaser

Microglial proliferation and activation is a hallmark of Alzheimer’s disease. Olmos-Alonso *et al.* show that microglial proliferation in Alzheimer’s disease tissue correlates with overactivation of the colony-stimulating factor 1 receptor (CSF1R) pathway. CSF1R blockade arrests microglial proliferation and activation in a mouse model of Alzheimer-like pathology and slows disease progression.

## Graphical abstract

**Figure:**
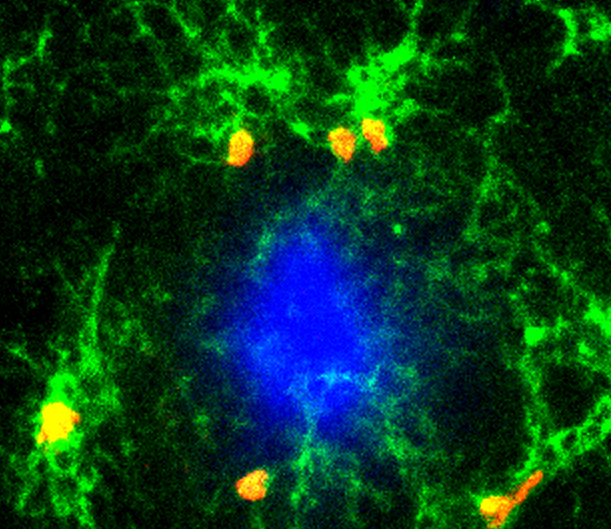
Microglial proliferation and activation is a hallmark of Alzheimer’s disease. Olmos-Alonso *et al.* show that microglial proliferation in Alzheimer’s disease tissue correlates with overactivation of the colony-stimulating factor 1 receptor (CSF1R) pathway. CSF1R blockade arrests microglial proliferation and activation in a mouse model of Alzheimer-like pathology and slows disease progression.

## Introduction

The neuropathology of Alzheimer’s disease shows a robust innate immune response characterized by the presence of activated microglia, with increased or *de novo* expression of diverse macrophage antigens ( Akiyama *et al.* , 2000 ; Edison *et al.* , 2008 ), and production of inflammatory cytokines ( Dickson *et al.* , 1993 ; Fernandez-Botran *et al.* , 2011 ). Evidence indicates that non-steroidal anti-inflammatory drugs (NSAIDs) protect from the onset or progression of Alzheimer’s disease ( Hoozemans *et al.* , 2011 ), suggestive of the idea that inflammation is a causal component of the disease rather than simply a consequence of the neurodegeneration. In fact, inflammation ( Holmes *et al.* , 2009 ), together with tangle pathology ( Nelson *et al.* , 2012 ) or neurodegeneration-related biomarkers ( Wirth *et al.* , 2013 ) correlate better with cognitive decline than amyloid-β accumulation, but the underlying mechanisms of the sequence of events that contribute to the clinical symptoms are poorly understood. The contribution of inflammation to disease pathogenesis is supported by recent genome-wide association studies, highlighting immune-related genes such as *CR1* ( Jun *et al.* , 2010 ), *TREM2* ( Guerreiro *et al.* , 2013 ; Jonsson *et al.* , 2013 ) or *HLA-DRB5–HLA-DRB1* in association with Alzheimer’s disease ( European Alzheimer’s Disease *et al.* , 2013 ). Additionally, a growing body of evidence suggests that systemic inflammation may interact with the innate immune response in the brain to act as a ‘driver’ of disease progression and exacerbate symptoms ( Holmes *et al.* , 2009 , 2011 ).

Microglial cells are the master regulators of the neuroinflammatory response associated with brain disease ( Gomez-Nicola and Perry, 2014 *a* , *b* ). Activated microglia have been demonstrated in transgenic models of Alzheimer’s disease ( LaFerla and Oddo, 2005 ; Jucker, 2010 ) and have been recently shown to dominate the gene expression landscape of patients with Alzheimer’s disease ( Zhang *et al.* , 2013 ). Recently, microglial activation through the transcription factor PU.1 has been reported to be capital for the progression of Alzheimer’s disease, highlighting the role of microglia in the disease-initiating steps ( Gjoneska *et al.* , 2015 ). Results from our group, using a murine model of chronic neurodegeneration (prion disease), show large numbers of microglia with an activated phenotype ( Perry *et al.* , 2010 ) and a cytokine profile similar to that of Alzheimer’s disease ( Cunningham *et al.* , 2003 ). The expansion of the microglial population during neurodegeneration is almost exclusively dependent upon proliferation of resident cells ( Gomez-Nicola *et al.* , 2013 , 2014 *a* ; Li *et al.* , 2013 ). An increased microglial proliferative activity has also been described in a mouse model of Alzheimer’s disease ( Kamphuis *et al.* , 2012 ) and in post-mortem samples from patients with Alzheimer’s disease ( Gomez-Nicola *et al.* , 2013 , 2014 *b* ). This proliferative activity is regulated by the activation of the colony stimulating factor 1 receptor (CSF1R; Gomez-Nicola *et al.* , 2013 ). Pharmacological strategies inhibiting the kinase activity of CSF1R provide beneficial effects on the progression of chronic neurodegeneration, highlighting the detrimental contribution of microglial proliferation ( Gomez-Nicola *et al.* , 2013 ). The presence of a microglial proliferative response with neurodegeneration is also supported by microarray analysis correlating clinical scores of incipient Alzheimer’s disease with the expression of *Cebpa* and *Spi1* (PU.1), key transcription factors controlling microglial lineage commitment and proliferation ( Blalock *et al.* , 2004 ). Consistent with these data, *Csf1r* is upregulated in mouse models of amyloidosis ( Murphy *et al.* , 2000 ), as well as in human post-mortem samples from patients with Alzheimer’s disease ( Akiyama *et al.* , 1994 ). Although these ideas would lead to the evaluation of the efficacy of CSF1R inhibitors in Alzheimer’s disease, we have little evidence regarding the level of microglial proliferation in Alzheimer’s disease or the effects of CSF1R targeting in animal models of Alzheimer’s disease-like pathology. In this study, we set out to define the microglial proliferative response in both human Alzheimer’s disease and a mouse model of Alzheimer’s disease-like pathology, as well as the activation of the CSF1R pathway. We provide evidence for a consistent and robust activation of a microglial proliferative response, associated with the activation of CSF1R. We provide proof-of-target engagement and efficacy of an orally available CSF1R inhibitor (GW2580), which inhibits microglial proliferation and partially prevents the pathological progression of Alzheimer’s disease-like pathology, supporting the evaluation of CSF1R-targeting approaches as a therapy for Alzheimer’s disease.

## Materials and methods

### Study design

#### Sample size

The most suitable statistical test for our samples and experiments, which reach the assumptions of normality and homoscedasticity is the one-way or two-way ANOVA. After performing power calculations, to achieve a significant difference of *P <* 0.05, in light of a retrospective analysis of our previous results ( Gomez-Nicola *et al.* , 2013 , 2014 *a* , *b* ), we needed a minimum of *n =* 4 (immunohistochemistry, RT-PCR) and *n =* 6 (behaviour) to reach a power between 0.80–0.90, depending on the specific experimental conditions. The calculations are the customary ones based on normal distributions and were performed following statistical advice from the Research design and methodology Department of the University of Southampton.

#### Data inclusion and exclusion

For RNA expression experiments performed in human post-mortem samples, we excluded samples showing a low yield and quality of recovered RNA, judged as having a difference in the expression of the four selected housekeeping genes higher than 5 Ct values and poor cross-correlation when compared to the other samples. No samples were excluded from the experiments using APP/PS1 mice.

#### Randomization and blinding

The experiments were designed in compliance with the ARRIVE guidelines, including control groups for all experiments, randomizing the procedures and applying double-blinded analysis when possible.

### Animals

APPswe/PSEN1dE9 mice (APP/PS1) on a C57BL/6 background were originally obtained from The Jackson Laboratory ( McClean *et al.* , 2011 ). Heterozygous males were bred with wild-type female C57BL/6J (Harlan) at our local facilities. APP/PS1/Macgreen (c-fms EGFP) mice maintained at our local facilities, after breeding c-fms EGFP mice ( Sasmono *et al.* , 2003 ) with the APP/PS1 line, allowing the Macgreen transgene to be expressed in heterozygosis. Offspring were ear punched and genotyped using PCR with primers specific for the APP-sequence (forward: GAATTCCGACATGACTCAGG, reverse: GTTCTGCTGCATCTTGGACA). Mice not expressing the transgene were used as wild-type controls. Mice were housed in groups of 4 to 10, under a 12-h light/12-h dark cycle at 21°C, with food and water *ad libitum* .

For the evaluation of the effects of treatment with GW2580, mice were 6 months of age when treatment began ( *n =* 6–8). Mice were fed with a control diet (RM1) or a diet containing GW2580 [Modified LabDiet® PicoLab EURodent Diet 14%, 5L0W (5LF2) with 0.1% (1000 ppm) GW2580 (LC Laboratories); TestDiet] for 3 months, before behavioural tasks were conducted. Alternatively, to test the effects of increasing doses of GW2580 on microglial survival, GW2580 (LC Laboratories) was suspended in 0.5% hydroxypropylmethylcellulose and 0.1% Tween 80 and was dosed orally at 0.2 ml per mouse (75 mg/kg), daily for five consecutive days to wild-type mice. Mice weight was monitored during all experiments. Mice received one injection of intraperitoneal bromodeoxyuridine (BrdU) (Sigma-Aldrich; 7.5 mg/ml, 0.1 ml/10 g weight in sterile saline), 1 day before the end of the experiment. All procedures were performed in accordance with UK Home Office licensing.

### Post-mortem samples of Alzheimer’s disease

For immunohistochemical analysis, human brain autopsy tissue samples (temporal cortex, paraffin-embedded, formalin-fixed, 96% formic acid-treated, 6-µm sections) from the National CJD Surveillance Unit Brain Bank (Edinburgh, UK) were obtained from cases of Alzheimer’s disease (five females and five males, age 58–76) or age-matched controls (four females and five males, age 58–79), in whom consent for use of autopsy tissues for research had been obtained. All cases fulfilled the criteria for the pathological diagnosis of Alzheimer’s disease. Ethical permission for research on autopsy materials stored in the National CJD Surveillance Unit was obtained from Lothian Region Ethics Committee.

For mRNA analysis, human brain autopsy tissue samples (temporal cortex, fresh-frozen tissue) were obtained from the Human Tissue Authority licensed South West Dementia Brain Bank, University of Bristol (UK). Samples were selected from Alzheimer’s disease cases and age-matched controls ( Supplementary Table 1 ). Ethical permission for research on autopsy materials stored in the South West Dementia Brain Bank was obtained from Local Ethics Committee.

### Behavioural tests

APP/PS1 or wild-type mice treated with control (RM1) or GW2580 diet, as stated earlier, were tested on behavioural tasks at 9 months of age (3 months into the diet): open-field locomotor and exploratory activity and burrowing activity (sickness behaviour). For the behavioural analysis, *n =* 8–21 was used.

The open-field tests were carried out using activity monitor software (Med Associated Inc.). The mice were placed in individual cages of 27 × 27 × 0.3 cm for a period of 5 min, to further analyse the total distance travelled (cm) and the number of rears (vertical counts), using the average speed as an internal control of the mouse motor abilities, during the test period (5 min). For measuring anxiety-related behaviour, differential exploratory activity (distance travelled or number of entries) was analysed in a residual and an open zone, using activity monitor software (Med Associated Inc.), as previously described ( Rothman *et al.* , 2012 ).

For burrowing behaviour, plastic cylinders, 20-cm long and 6.8-cm in diameter were filled with 190 g of normal diet food pellets and placed in individual mouse cages. Mice were placed individually in the cages overnight, weighting the remaining pellets at the end of each session, and calculating the amount displaced (‘burrowed’). The mice were returned then to their home cage. Body weights and diet consumption of all mice were monitored during the course of the experiment.

Discrete trial spontaneous alternation in the T-maze was performed as previously described ( Deacon and Rawlins, 2006 ). The apparatus for this test consisted of a grey T-shaped maze with 30 × 10 × 29-cm arms, with a central partition extending 7 cm into the start arm from the back of the maze, and two guillotine doors each having the potential to block off the left or right goal arms. Mice were placed in the start arm of the maze (facing the wall) and allowed to make a choice to enter the left or right goal arm. Following their choice, they were then enclosed in that arm for 30 s to facilitate habituation by sliding down the appropriate guillotine door. Mice were then taken out of the maze, the central partition removed and guillotine doors reopened. Mice were once again placed in the start arm and allowed to make another free choice of either goal arm. Whether or not the mouse alternated was noted, with a score of 1 given if the mouse visited the other arm reflecting exploratory behaviour and memory of the first choice on its second trial, and a score of 0 given if the mouse went to the same arm on its second trial. The alternation ratio was obtained as the mean of the 20 scores. If animals did not move in 90 s or less these were considered failed trials and were not scored; however, these did not account for >5% of all trials and there was no effect of treatment or genotype for missed trials ( *P* = 0.97). The test was performed four times a day for 5 days (a total of 20 trials) with an average spacing of 2 h in between each trial.

### Immunohistochemistry

Coronal hippocampal sections were cut from paraformaldehyde-fixed, frozen or fresh brains. Mice perfusion, tissue processing and immunohistochemical analysis was performed as previously described ( Gomez-Nicola *et al.* , 2008 , 2013 ), using the following primary antibodies: rabbit anti-Iba1 (Wako), mouse anti-human Ki67 (Dako), mouse anti-BrdU (DSHB), mouse anti-amyloid-β (6E10; Covance), mouse anti-synaptophysin (SY38; Merck Millipore), rabbit anti-CSF1R (Santa Cruz Biotechnologies) and rabbit anti-PU.1 (Cell Signaling). Following primary antibody incubation, the sections were washed and incubated with the appropriate biotinylated secondary antibody (Vector Labs), and/or with the appropriate Alexa 405, 488 or 568 conjugated secondary antibody or streptavidin (Molecular Probes). For co-labelling of Iba1 and Ki67 in human tissue, following primary antibody, sections were incubated with an anti-rabbit biotinylated secondary antibody (for Iba1 detection) and the ImmPRESS-AP Anti-Mouse (alkaline phosphatase) Polymer Detection Kit (for Ki67 detection), as previously described ( Gomez-Nicola and Boche, 2015 ; Gomez-Nicola and Perry, 2016 ). For light microscopy, the sections were visualized using 3,3’-diaminobenzidine (DAB) precipitation or BCIP/NBT AP reaction, in a Leica CTR 5000 microscope, coupled to a Leica DFC300FX microscope camera. For PU.1 visualization, the DAB signal was enhanced with 0.05% nickel ammonium sulphate, producing a black precipitate. After immunofluorescence labelling, nuclei were visualized by DAPI staining and the sections were mounted with Mowiol/DABCO (Sigma-Aldrich) mixture. The sections were visualized on a Leica TCS-SP5 confocal system, coupled to a Leica CTR6500 microscope.

The general immunohistochemistry protocol was modified for the detection of BrdU, adding a DNA denaturation step with 2 N HCl (30 min, 37 **°** C), as previously described ( Gomez-Nicola *et al.* , 2011 , 2013 ). For the detection of amyloid-β plaques (6E10), sections were pretreated with 95% formic acid (Sigma-Aldrich) for 10 min at room temperature. All other histological stains (i.e. Congo red) were done according to standard laboratory procedures.

The protocol used for immunohistochemistry on human sections was a modification of the general protocol (DAB + alkaline phosphatase), with antigen unveiling in citrate buffer being performed for 25 min, as previously described ( Gomez-Nicola *et al.* , 2014 *b* ).

### Golgi-Cox staining

A subgroup of APP/PS1 or wild-type mice treated with control (RM1) or GW2580 diet ( *n =* 4), as stated before, were deeply anaesthetized with sodium pentobarbital and then transcardially perfused with artificial CSF. Brains were then rapidly dissected and sliced with a vibrating microtome (200 µm; Leica). The hippocampal slices were incubated in rapid Golgi-Cox solutions, following manufacturer’s instructions (FD Rapid Golgi Stain Kit, FD Neurotechnologies) **.** The slices were infused with a solution containing potassium dichromate, potassium chromate and mercuric chloride for 2 weeks, to be further developed into the Golgi-Cox staining on free-floating plates. The slices were mounted onto gelatinized slides, dried, dehydrated, cleared with xylene and mounted with DPX. Golgi-treated slices were analysed with a Leica CTR 5000 microscope, coupled to a Leica DFC300FX microscope camera. For the dendritic linear spine density, Golgi-Cox-labelled apical dendritic processes of CA1 neurons were analysed.

### Quantification and image analysis

The quantification of antigen positive cells (i.e. PU.1 ^+^ ) in the cerebral cortex ( *n =* 4 fields/mouse, *n =* 4–8 mice/group) was performed after DAB immunohistochemistry. The number of double positive cells (i.e. Iba1 ^+^ BrdU ^+^ ) in the specific area ( *n =* 4 fields/mouse, *n =* 4–8mice/group) was performed after double immunofluorescence or double immunohistochemistry with DAB/AP. Data were represented as number of positive cells/mm ^2^ . The quantification of antigen-positive cells (i.e. Iba1 ^+^ BrdU ^+^ or Iba1 ^+^ ) in human brains was performed in the white or grey matter of the temporal cortex after DAB/AP immunohistochemistry ( *n =* 20 fields/brain, *n =* 9–10 brains/group). The quantification of the distribution of microglial cells (PU.1 ^+^ ) around amyloid-β plaques was performed with an adapted version of the Sholl analysis, modified from Frautschy *et al.* (1998) . Briefly, we analysed all amyloid-β plaques visible with Congo red staining ( *n =* 4–6 sections/mouse, *n =* 4–8 mice/group), tracing concentric circles starting from the diameter of each individual plaque and setting radius step size at 20 μm. The final radius was set when (i) an individual plaque came into contact with a neighbouring plaque; (ii) cell density reached wild-type levels; or (iii) a border of the tissue was reached. PU.1 ^+^ cells density (cells/mm ^2^ ) contained within each circle was quantified, considering that cells falling at the interphase of two radii were counted as belonging to the section containing >50% of the cell. The quantification of the intensity of signal (i.e. synaptophysin) was performed after immunofluorescence, and presented as per cent stained area. The quantification of enhanced green fluorescent protein (EGFP) intensity per cell in APP/PS1/Macgreen mice was performed on confocal stacks, using a constant sampled area. All quantifications were performed with the help of the ImageJ image analysis software.

### Analysis of gene expression by reverse transcriptase PCR

APP/PS1 or wild-type mice treated with control (RM1) or GW2580 diet ( *n =* 4–6/group) were processed to obtain samples from the cortex by dissection under a microscope, after intracardiac perfusion with heparinized 0.9% saline. RNA was extracted using TRIzol® (Life Technologies), quantified using Nanodrop (Thermo Scientific), and reverse transcribed using the iScript™ cDNA Synthesis Kit (Bio-Rad) following manufacturer’s instructions, after checking its integrity by electrophoresis in a 2% agarose gel. cDNA libraries were analysed by quantitative polymerase chain reaction (PCR) using the iTaq™ Universal SYBR® Green supermix (Bio-Rad) and the following custom designed gene-specific primers (Sigma-Aldrich): csf1 (NM_007778.4; FW, agtattgccaaggaggtgtcag, RV, atctggcatgaagtctccattt), il34 (NM_001135100.1; FW, ctttgggaaacgagaatttggaga, RV, gcaatcctgtagttgatggggaag), csf1r (NM_001037859.2; FW, gcagtaccaccatccacttgta, RV, gtgagacactgtccttcagtgc), pu.1 (NM_011355.1; FW, cagaagggcaaccgcaagaa, RV, gccgctgaactggtaggtga), c/ebpa (NM_007678.3; FW, agcttacaacaggccaggtttc, RV, cggctggcgacatacagtac), runx1 (NM_001111021; FW, caggcaggacgaatcacact, RV, ctcgtgctggcatctctcat), irf8 (NM_008320; FW, cggggctgatctgggaaaat, RV, cacagcgtaacctcgtcttc), il1b (NM_008361.3; FW, gaaatgccaccttttgacagtg, RV, tggatgctctcatcaggacag), tgfb (NM_011577; FW, tgtacggcagtggctgaacc, RV, cgtttggggctgatcccgtt), il6 (NM_031168; FW, tagtccttcctaccccaatttcc, RV, ttggtccttagccactccttc) or igf1 (NM_010512; FW, agatctgcctctgtgacttcttga, RV, agcctgtgggcttgttgaagt). Quality of the primers and the PCR reaction were evaluated by electrophoresis in a 1.5% agarose gel, checking the PCR product size. Data were analysed using the 2-ΔΔCt method with Primer Opticon 3 software, using *Gapdh* (NM_008084.2; FW, tgaacgggaagctcactgg, RV, tccaccaccctgttgctgta) as a housekeeping gene.

Frozen samples from Alzheimer’s disease cases or age-matched controls ( Supplementary Table 1 ) were processed for RNA extraction and quantitative PCR analysis. RNA was extracted using the RNAqueous-Micro Kit (Life Technologies), quantified using Nanodrop (Thermo Scientific), reverse transcribed using the iScript cDNA Synthesis Kit (Bio-Rad), following the manufacturer’s instructions, after checking its integrity by electrophoresis on a 1.8% agarose gel. Low quality and purity RNA samples were excluded from consequent experimentation. cDNA libraries were analysed by quantitative PCR using 96-wells custom-designed TaqMan® array plates with the 7500 Real-Time PCR system (Applied Biosystems). Quality of the PCR reaction end product was evaluated by electrophoresis in a 1.5% agarose gel. Raw CT data were obtained from the SDS v.2.0.6 software and normalized to the normalization factor (geometric mean of four housekeeping genes; *GAPDH* , *HPRT* , *18S* and *GUSB* ) using the 2-ΔΔCT method.

### Antibody arrays

APP/PS1 or wild-type mice treated with control (RM1) or GW2580 diet ( *n =* 4–6/group) were processed to obtain samples from the cortex by dissection under a microscope, after intracardiac perfusion with heparinized 0.9% saline. Tissues were homogenized in RIPA buffer and protein concentration was quantified with a BCA protein assay kit (Pierce). The concentration of 40 mouse cytokines and chemokines was analysed with Mouse Quantibody Cytokine Arrays Q5 (QAM-CYT-5; Raybiotech) by the Raybiotech testing service, according to manufacturer’s instructions. The concentration of every cytokine was quantified using internal protein standards, discarding data not reaching the detection threshold (LOD, limit of detection). Data were represented as fold-change of APP/PS1 + RM1 versus wild-type + RM1 and APP/PS1 + GW2580 versus wild-type + GW2580.

### Multiplex analysis of soluble amyloid-β _38_ , amyloid-β _40_ and amyloid-β _42_

Protein samples obtained and quantified as above were analysed with an Aβ V-plex triple ultra-sensitive assay kit according to the manufacturer’s instructions (Meso Scale Discovery). Standards (amyloid-β _1–38_ , amyloid-β _1–40_ and amyloid-β _1–42_ ) and samples (diluted 1:20) were added to the 96-well plates, incubated, washed, and read in a Sector Imager plate reader (Meso Scale Discovery) immediately after addition of the Meso Scale Discovery read buffer. Amyloid-β concentrations were calculated with reference to the standard curves and expressed as nanograms per millilitre.

### Statistical analysis

Data were expressed as mean ± standard error of the mean (SEM) and analysed with the GraphPad Prism 5 software package (GraphPad Software). For all datasets, normality and homoscedasticity assumptions were reached, validating the application of the two-way ANOVA (two variables were analysed in all cases), followed by the Tukey *post hoc* test for multiple comparisons. Relative gene expression data from human samples was analysed using a two-tailed Fisher *t* -test. Correlation of relative gene expression in human samples to Braak stage was tested using the Kendall tau-b rank correlation test included in the SPSS analytic software (v.17; IBM). Differences were considered significant for *P <* 0.05.

## Results

### Microglial proliferation is increased in Alzheimer’s disease and correlates with disease severity

**Figure 1:**
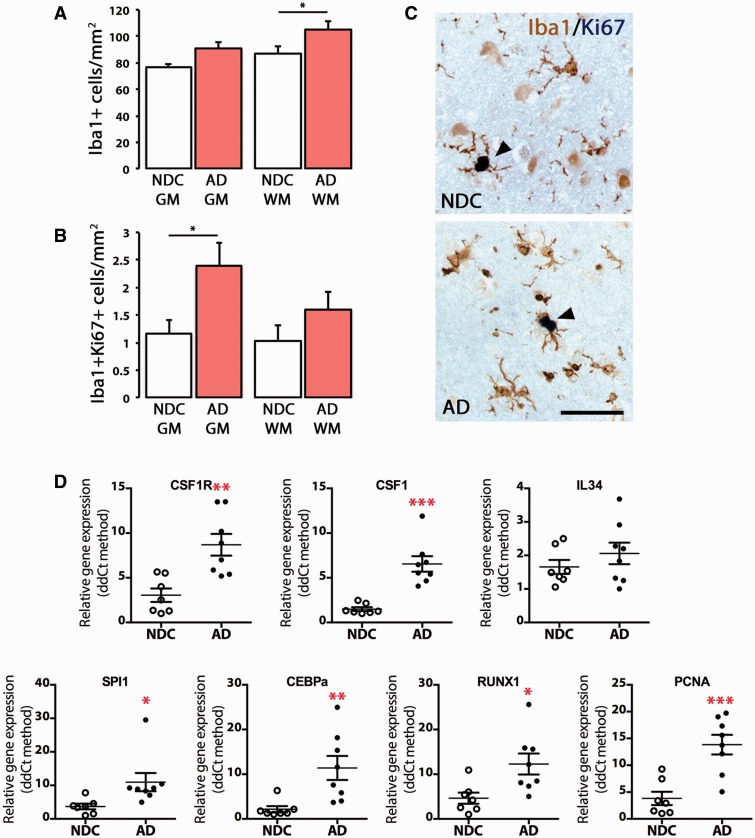
**Characterization of the microglial proliferative response in Alzheimer’s disease.** ( **A–C** ) Immunohistochemical analysis and quantification of the number of total microglial cells (Iba1 ^+^ ; **A** ) or proliferating microglial cells (Iba1 ^+^ Ki67 ^+^ ; **B** ) in the grey (GM) and white matter (WM) of the temporal cortex of Alzheimer’s disease cases (AD) and age-matched non-demented controls (NDC). ( **C** ) Representative pictures of the localization of a marker of proliferation (Ki67, dark blue) in microglial cells (Iba1 ^+^ , brown) in the grey matter of the temporal cortex of non-demented controls or Alzheimer’s disease cases. ( **D** ) RT-PCR analysis of the mRNA expression of *CSF1R* , *CSF1* , *IL34* , *SPI1* (PU.1), *CEBPA* , *RUNX1* and *PCNA* in the temporal cortex of Alzheimer’s disease cases and age-matched non-demented controls. Expression of mRNA represented as mean ± SEM and indicated as relative expression to the normalization factor (geometric mean of four housekeeping genes; *GAPDH* , *HPRT* , *18S* and *GUSB* ) using the 2-ΔΔCT method. Statistical differences: * *P <* 0.05, ** *P <* 0.01, *** *P <* 0.001. Data were analysed with a two-way ANOVA and a *post hoc* Tukey test ( **A** and **B** ) or with a two-tailed Fisher *t* -test ( **D** ). Scale bar in **C** = 50 µm.

**Figure 2:**
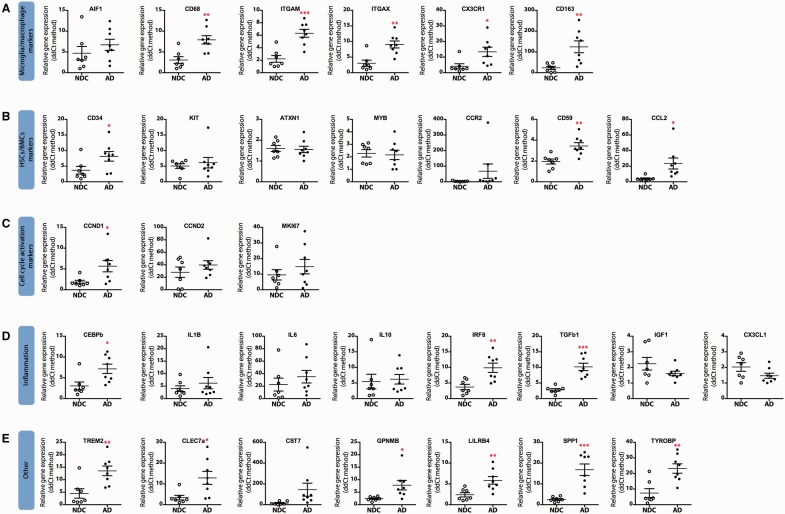
**Gene expression analysis in human post-mortem Alzheimer’s disease cases and age-matched controls.** Samples from Alzheimer’s disease (filled circles) cases or age-matched controls (NDC, open circles) were analysed by quantitative PCR for the expression of microglia/macrophage markers ( **A** ), hematopoietic stem cell/bone marrow-derived cell (HSCs/BMCs) markers ( **B** ), cell cycle activation markers ( **C** ), inflammation markers ( **D** ) or other ( **E** ). Samples were analysed using custom-designed TaqMan ^®^ array plates with the 7500 Real-Time PCR system (Applied Biosystems). Expression of mRNA represented as mean ± SEM and indicated as relative expression to the normalization factor (geometric mean of four housekeeping genes; *GAPDH* , *HPRT* , *18S* and *GUSB* ) using the 2-ΔΔCT method. Statistical differences: * *P <* 0.05, ** *P <* 0.01, *** *P <* 0.001. Data were analysed with a two-tailed Fisher *t* -test.

Although an increased microglial response has been documented in Alzheimer’s disease ( Bondolfi *et al.* , 2002 ; Gomez-Nicola and Perry, 2014 *a* , *b* ), we identified the need for a better understanding of microglial proliferation and the expression of the potential pro-mitogenic components in human tissue. The analysis of post-mortem cases of Alzheimer’s disease (temporal cortex; Supplementary Table 1 ) evidenced an increase in the total number of microglial cells in cortical grey matter (118% versus non-demented controls, not significant) and white matter (120% versus non-demented controls, *P <* 0.05) ( Fig. 1 A). We also observed an increased microglial proliferation ( Fig. 1 B and C), mostly evident in the grey matter (207% versus non-demented controls, *P <* 0.05) than in the white matter (155% versus non-demented controls, not significant). At the gene expression level, we found an elevated expression of the main components of the CSF1R pro-mitogenic pathway ( *CSF1R* , *CSF1* , *SPI1* , *CEBPA* , *RUNX1* ; Fig. 1 D). Several microglial markers are upregulated in Alzheimer’s disease, while the majority of hematopoietic stem cell or bone marrow-derived cell markers were found to be unchanged when compared with non-demented controls, with *CD34* , *CD59* and *CCL2* being upregulated ( Fig. 2 A and B). We also evidenced an increased expression of markers related to cell proliferation, such as *PCNA* , ( Figs 1 D and 2 C) and the activation of an inflammatory response characterized by *TGFB* expression and low levels of proinflammatory cytokines ( *IL1B* , *IL6* ), consistent with previously reported findings ( Gomez-Nicola and Perry, 2014 *a* , *b* ) ( Fig. 2 D and E). The analysis of the correlation of gene expression with the Braak score evidenced a significant association of disease severity with several markers of microglial cells [ *ITGAM* (CD11b), *ITGAX* (CD11c), *CD68* , *CX3CR1* ], cell proliferation ( *CEBPA* , *CSF1* , *PCNA* , *SPI1* ) and inflammation ( *CCL2* , *CEBPB* , *CX3CL1* , *TGFB* or *TREM2* ) ( Supplementary Table 2 ). The expression of markers of perivascular macrophages or bone marrow-derived cells ( *CD163* , *CCR2* , *CD34* , *CD59* ) correlated with Alzheimer’s disease severity, while we found no correlation with hematopoietic stem cell markers ( *KIT* , *MYB* , *ATXN1* ; Supplementary Table 2 ).

These findings highlight the relevance of microglial proliferation and activation for the progression of Alzheimer’s disease, and support the study of these mechanisms in animal models of Alzheimer’s disease-like pathology.

### Increased microglial proliferation and CSF1R activity are closely associated with the progression of Alzheimer’s disease-like pathology

**Figure 3:**
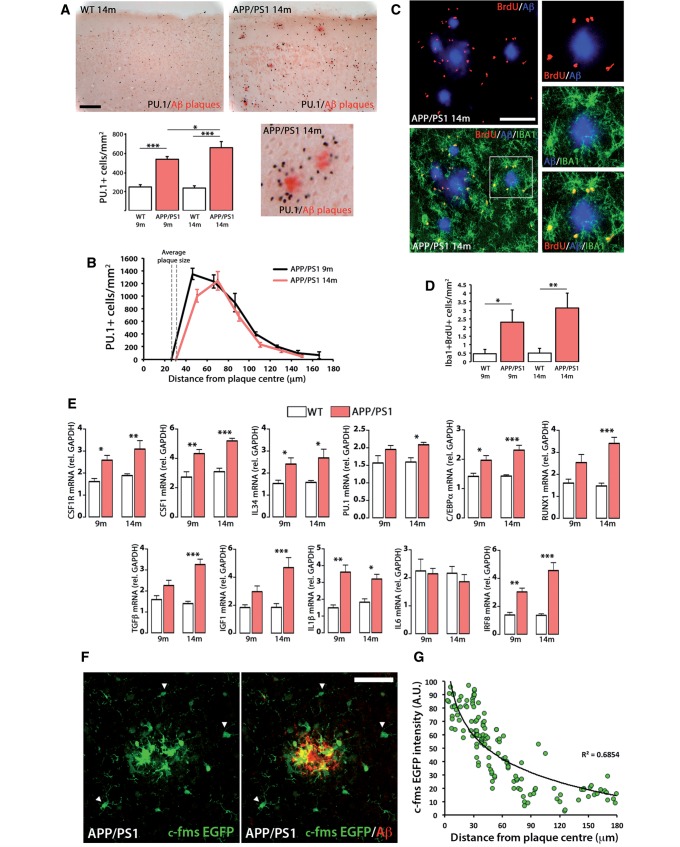
**Characterization of the microglial proliferative response in a mouse model of Alzheimer’s disease-like pathology (APP/PS1).** ( **A** ) Immunohistochemical analysis and quantification of the number of total microglial cells (PU.1 ^+^ ; black) in the cortex of APP/PS1 and wild-type mice at 9 and 14 months of age. Amyloid-β plaques are shown in red (Congo Red). Number of microglia represented as mean ± SEM of PU.1 ^+^ cells/mm ^2^ . ( **B** ) Analysis of the spatial distribution of microglial cells (PU.1 ^+^ ) around amyloid-β plaques in the cortex of APP/PS1 mice at 9 and 14 months of age, using an adapted version of the Sholl analysis (see ‘Materials and methods’ section). Number of microglia represented as mean ± SEM of PU.1 ^+^ cells/mm ^2^ . ( **C** and **D** ) Analysis and quantification of microglial proliferation (Iba1 ^+^ BrdU ^+^ , green and red, respectively; **C** ) by triple immunofluorescence analysed by confocal microscopy. Amyloid-β plaques are shown in blue (6E10). ( **D** ) Microglial proliferation represented as mean ± SEM of Iba1 ^+^ BrdU ^+^ cells/mm ^2^ . ( **E** ) RT-PCR analysis of the mRNA expression of *Csf1r* , *Csf1* , *Il34* , *Spi1* (PU.1), *Cebpa* , *Runx1* , *Tgfb* , *Igf1* , *Il1b* , *Il6* and *Irf8* in the cortex of APP/PS1 and wild-type (WT) mice at 9 and 14 months of age. Expression of mRNA represented as mean ± SEM and indicated as relative expression compared to the housekeeping gene ( *Gapdh* ) using the 2-ΔΔCT method. ( **F** ) Immunofluorescent analysis of the expression of EGFP (green) driven by the *Csf1r* promoter in APP/PS1/Macgreen mice, around amyloid-β plaques in the cortex of APP/PS1 mice at 14 months of age. Amyloid-β (6E10) is shown in red. Arrowheads indicate microglia with low CSF1R expression. ( **G** ) Correlation analysis of the expression of EGFP in individual cells in APP/PS1/Macgreen mice with the distance to amyloid-β plaques. Statistical differences: * *P <* 0.05, ** *P <* 0.01, *** *P <* 0.001. Data were analysed with a two-way ANOVA and a *post hoc* Tukey test ( **A** , **D** and **E** ). Scale bars: **A** = 100 μm, **C** and **F** = 50 μm.

We investigated the proliferative dynamics of microglial cells and the expression of the components of the CSF1R pathway in a relevant model of Alzheimer’s disease-like pathology (APPswe, PSEN1dE9; APP/PS1). While microglial accumulation around amyloid-β plaques has been documented ( Brawek *et al.* , 2014 ), we took advantage of the specificity of PU.1 expression in microglia to quantify microglial numbers, as it allows an accurate identification of microglial nuclei ( Fig. 3 A) ( Gomez-Nicola *et al.* , 2013 , 2014 *a* ). Microglial numbers increase progressively in APP/PS1 mice (from 9 to 14 months of age), when compared to wild-type littermates, accumulating in the vicinity of amyloid-β plaques ( Fig. 3 A). We observed that while the number of plaques in APP/PS1 mice increases with age (APP/PS1 9 months = 4.41 ± 0.39 versus APP/PS1 14 months = 7.25 ± 1.82 plaques/mm ^2^ ), neither their size nor the pattern of microglial distribution change ( Fig. 3 B). Microglial proliferation was evidenced by the incorporation of BrdU in Iba1 ^+^ cells, analysed by confocal microscopy ( Fig. 3 C). The number of Iba1 ^+^ /BrdU ^+^ cells increases progressively in APP/PS1 mice ( Fig. 3 D), in close association with amyloid-β plaques ( Fig. 3 C).

Further analysis of gene expression showed upregulation of the main components of the CSF1R pathway in APP/PS1 brains, with some genes being upregulated from 9 months onwards ( *Csf1* , *Il34* , *Csf1r* , *Cebpa* ) and others from 14 months [ *Spi1* (Pu.1), *Runx1* ] ( Fig. 3 E). These changes coincide with the upregulation of key inflammatory genes associated with microglial activation ( Fig. 3 E).

We next analysed the localization of CSF1R in APP/PS1/Macgreen mice (c-fms EGFP), as an optimal reporter line for CSF1R expression, as EGFP is driven by the *Csf1r* promoter (c-fms) ( Sasmono *et al.* , 2003 ). APP/PS1/Macgreen mice showed a differential increased expression levels of this receptor in microglial cells associated to amyloid-β plaques, when compared to microglia distal to plaques ( Fig. 3 F), with EGFP levels and distance to plaque centre showing inversed correlation ( Fig. 3 G). This correlation can also be observed when using CSF1R immunostaining ( Supplementary Fig. 1 ) and it is suggestive of an association of CSF1R levels with the observed proliferative response.

### Pharmacological targeting of CSF1R activation with an orally-available inhibitor blocks microglial proliferation in APP/PS1 mice

As CSF1R is likely to drive microglial proliferation in APP/PS1 mice, we targeted the activation of CSF1R with a selective inhibitor (GW2580) from 6 to 9 months of age, to evaluate target engagement and efficacy of a CSF1R-inhibitory approach ( Fig. 5 ). To determine if microglia would be the only target of CSF1R inhibitors, we used APP/PS1/Macgreen mice as reporters of CSF1R expression. Using immunofluorescence and confocal microscopy we found no evidence of expression of CSF1R in neurons (NeuN ^+^ ) or astrocytes (GFAP ^+^ ) ( Supplementary Fig. 2 ), supporting that CSF1R expression is exclusive to microglia, as previously described ( Sasmono *et al.* , 2003 ; Erblich *et al.* , 2011 ; Gomez-Nicola *et al.* , 2013 ). While a dose of GW2580 of 75 mg/kg has been used in the past without causing significant changes in the survival of microglia ( Gomez-Nicola *et al.* , 2013 ), recent evidence supports that CSF1R inhibitors could cause the depletion of the microglial population ( Elmore *et al.* , 2014 ). Therefore, we assessed the effects of repeated increasing doses of GW2580 (75, 150, 300 mg/kg) on the survival of the microglial population ( Supplementary Fig. 3 ). We found that none of the doses of GW2580 caused any significant change in the total number of microglial cells ( Supplementary Fig. 3A and Supplementary Data ), supporting the continued use of the 75 mg/kg to maintain consistency with our previously published data ( Gomez-Nicola *et al.* , 2013 ).

**Figure 4:**
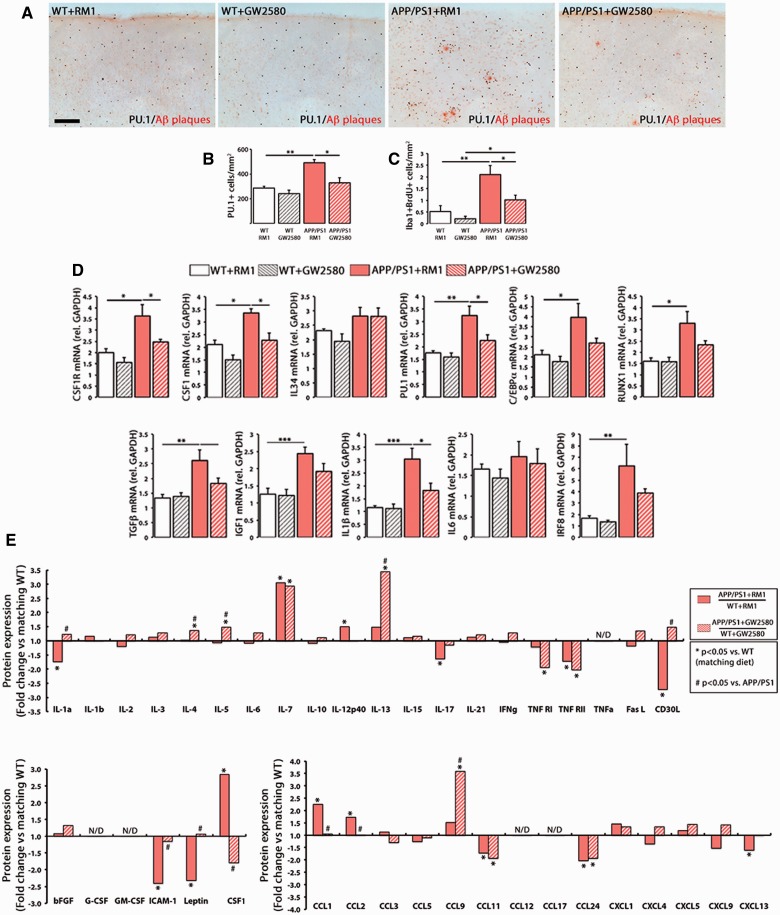
**Prolonged inhibition of CSF1R blocks microglial proliferation and rescues the inflammatory alterations of APP/PS1 mice.** ( **A–C** ) Immunohistochemical analysis and quantification of the number of total microglial cells (PU.1 ^+^ ; black, **A** and **B** ) and proliferating microglial cells (Iba1 ^+^ BrdU ^+^ , **C** ) in the cortex of APP/PS1 and wild-type mice at 9 months of age, after treatment for 3 months with a control diet (RM1) or a diet containing GW2580. Amyloid-β plaques are shown in red (Congo Red). Number of microglia represented as mean ± SEM of PU.1 ^+^ or Iba1 ^+^ BrdU ^+^ cells/mm ^2^ . ( **D** ) RT-PCR analysis of the mRNA expression of *Csf1r* , *Csf1* , *Il34* , *Spi1* (PU.1), *Cebpa* , *Runx1* , *Tgfb* , *Igf1* , *Il1b* , *Il6* and *Irf8* in the cortex of APP/PS1 and wild-type mice at 9 months of age, after treatment for 3 months with a control diet (RM1) or a diet containing GW2580. Expression of mRNA represented as mean ± SEM and indicated as relative expression compared to the housekeeping gene ( *Gapdh* ) using the 2-ΔΔCT method. ( **E** ) Quantification of protein concentration of 40 inflammatory mediators (grouped as cytokines, growth factors and chemokines) by Mouse Quantibody Cytokine Arrays (see ‘Materials and methods’ section), in samples from the cortex of APP/PS1 and wild-type mice at 9 months of age, after treatment for 3 months with a control diet (RM1) or a diet containing GW2580. Protein expression represented as fold change (+fold change = upregulation, −fold change = downregulation) of the corresponding APP/PS1 group (with RM1 or with GW2580) over its correspondent wild-type group. When protein concentration fell below the levels of detection of the assay for more than half of the samples, data are shown as N/D (not detectable). Statistical differences: * *P <* 0.05, ** *P <* 0.01. Data were analysed with a two-way ANOVA and a *post hoc* Tukey test. Scale bar: **A** = 100 μm.

Prolonged treatment with GW2580 caused a significant reduction in the number of microglial cells and a blockade of microglial proliferation in APP/PS1, when compared with APP/PS1 on control diet (RM1) ( Fig. 4 A–C). The treatment with the CSF1R selective inhibitor GW2580 did not cause depletion of the microglial population in either wild-type or APP/PS1 mice ( Fig. 4 A and B), suggesting specific targeting of microglial proliferation and not microglial survival. In line with these results, GW2580 downregulated the mRNA expression of *Csf1r* , *Csf1* , *Spi1* (PU.1), *Cebpa* and *Runx1* in APP/PS1 mice ( Fig. 4 D), all involved in controlling the pro-mitogenic programme. Some differences were observed in this dataset ( Fig. 4 D) compared to previous ones ( Fig. 3 E), suggesting significant variability in this model.

**Figure 5:**
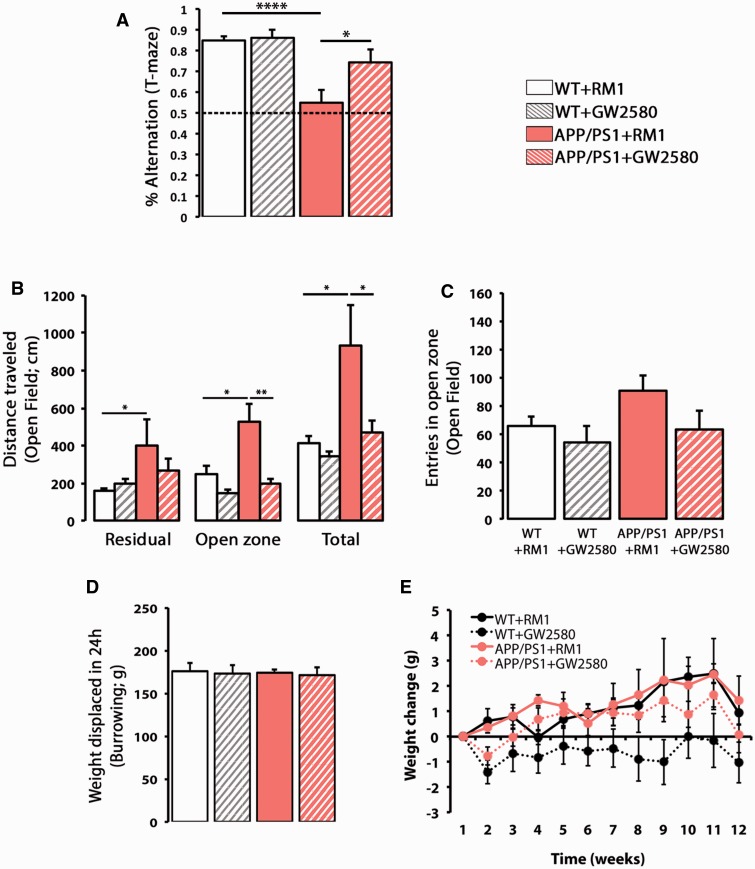
**CSF1R inhibition prevents behavioural deficits in APP/PS1 mice.** ( **A** ) Spontaneous alternation in the T-maze of APP/PS1 and wild-type mice at 9 months of age, after treatment for 3 months with a control diet (RM1) or a diet containing GW2580. Alternation was measured as % election of the alternative arm in the second test (short-term memory). ( **B** and **C** ) Analysis of the behaviour in the open field, measured as total distanced travelled ( **B** ) or number of entries in the open zone ( **C** ) of APP/PS1 and wild-type (WT) mice at 9 months of age, after treatment for 3 months with a control diet (RM1) or a diet containing GW2580. Exploratory activity was measured as distance travelled (cm) in the open field test, analysing the locomotor activity on an open zone versus residual zone as a correlate of anxiety. ( **D** ) Burrowing behaviour, a measure of sickness behaviour, was measured as weight displaced (g) off the tube in 24 h. ( **E** ) Analysis of the effect of the influence of genotype (wild-type versus APP/PS1) or diet (RM1 versus GW2580) on the average weekly weight change (relative to t = 0) mice at 9 months of age, after treatment for 3 months with a control diet (RM1) or a diet containing GW2580. Statistical differences: * *P <* 0.05, ** *P <* 0.01. Data were analysed with a two-way ANOVA and a *post hoc* Tukey ( **B–E** ) or Bonferroni ( **A** ) test.

To better understand the impact of the prevention of microglial proliferation in Alzheimer’s disease-like pathology we screened the expression of several inflammatory mediators at the mRNA level ( Fig. 4 D) and at the protein level using quantitative antibody arrays ( Fig. 4 E). Blockade of microglial proliferation induced a significant reduction in the expression of several inflammatory mediators at the mRNA level ( Fig. 4 D). CSF1R inhibition in APP/PS1 mice returned the expression of pro-inflammatory mediators such as IL1A, IL12, IL17, CD30L, CCL1, CCL2 or CXCL13 to wild-type levels, while upregulating the levels of anti-inflammatory cytokines such as IL4, IL5 or IL13 ( Fig. 4 E) or the chemokine CCL9 ( Fig. 4 E). Blockade of CSF1R prevented overexpression of CSF1 in APP/PS1, as well as the downregulation of ICAM1, leptin and TNFR1 ( Fig. 4 E), highlighting some potentially beneficial side-effects of CSF1R inhibition in microglial cells. Other molecules were unaffected by CSF1R inhibition, including IL7, TNFR2, CCL11 and CCL24 ( Fig. 4 E).

### CSF1R inhibition prevents the progression of Alzheimer’s disease-like pathology

The prolonged inhibition of CSF1R by GW2580 prevented the behavioural deficits observed in APP/PS1 mice ( Fig. 5 ). Blockade of microglial proliferation with GW2580 caused a significant recovery of the deficits in short-term memory observed in APP/PS1 mice, analysed as spontaneous alternation in the T-maze ( Fig. 5 A). The increased activity in the open-field task observed in APP/PS1 was prevented by the treatment with GW2580-containing diet ( Fig. 5 B). These effects were associated with the development of a hyperactive behaviour in APP/PS1 mice, rather than a change in anxiety-related behaviour, as evidenced by the lack of preferential use of the different zones of the arena ( Fig. 5 B) and the lack of differences in the number of entries in the open zone ( Fig. 5 C). A task associated with sickness behaviour (burrowing) was found to be unchanged in APP/PS1 mice ( Fig. 5 D). The treatment of wild-type or APP/PS1 mice with a GW2580-containing diet for 3 months did not cause any significant effects on weight gain, when compared with mice treated with a control diet (RM1) ( Fig. 5 E).

**Figure 6:**
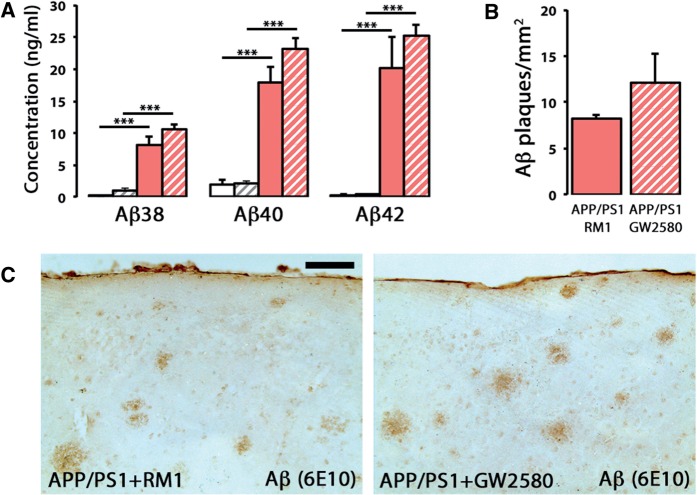
**CSF1R inhibition does not alter the levels of amyloid-β.** ( **A** ) Multiplexed analysis of the concentration of soluble amyloid-β _38_ , amyloid-β _40_ and amyloid-β _42_ in cortex homogenates of APP/PS1 and wild-type mice at 9 months of age, after treatment for 3 months with a control diet (RM1) or a diet containing GW2580. Soluble amyloid-β levels represented as mean ± SEM of concentration (ng/ml). ( **B** and **C** ) Immunohistochemical analysis and quantification of the number of amyloid-β plaques (6E10 ^+^ ; brown) in the cortex of APP/PS1 mice at 9 months of age, after treatment for 3 months with a control diet (RM1) or a diet containing GW2580. Number of amyloid-β plaques represented as mean ± SEM of 6E10 ^+^ plaques/mm ^2^ . Statistical differences: *** *P <* 0.001. Data were analysed with a two-way ANOVA and a *post hoc* Tukey test. Scale bar in **C** = 100 μm.

**Figure 7:**
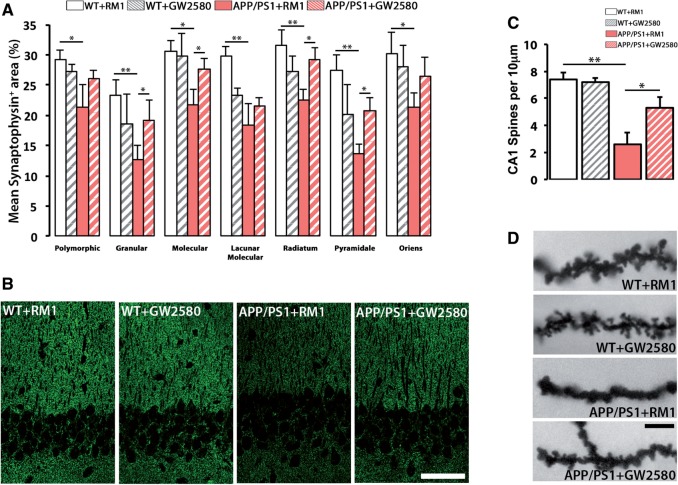
**CSF1R inhibition prevents synaptic degeneration in APP/PS1 mice.** ( **A** and **B** ) Immunohistochemical analysis and quantification of protein levels of synaptophysin in the hippocampus of APP/PS1 and wild-type (WT) mice at 9 months of age, after treatment for 3 months with a control diet (RM1) or a diet containing GW2580. Synaptophysin levels represented as mean ± SEM of %Synaptophysin ^+^ area ( **A** ). Representative confocal images are shown in **B** . ( **C** and **D** ) Analysis of spine density in the apical segment of hippocampal CA1 neurons of APP/PS1 and wild-type mice at 9 months of age, after treatment for 3 months with a control diet (RM1) or a diet containing GW2580. Representative images are shown in **D** . Statistical differences: * *P <* 0.05, ** *P <* 0.01. Data were analysed with a two-way ANOVA and a *post hoc* Tukey test. Scale bars: **B** = 50 μm, **D** = 10 μm.

One of the main pathological hallmarks in APP/PS1 mice, the deposition of amyloid-β plaques, was found unchanged after the treatment with GW2580, as evidenced by multiplex analysis of soluble amyloid-β _38_ , amyloid-β _40_ and amyloid-β _42_ ( Fig. 6 A), 6E10 immunostaining ( Figs 6 B and 7 C) and Congo Red staining (not shown), suggesting a requirement for microglial proliferation/activation to associate the amyloidogenic component with the behavioural decline observed in Alzheimer’s disease-like pathology. However, prolonged blockade of CSF1R prevented the synaptic degeneration observed in the hippocampus of APP/PS1 mice, as evidenced by a significant recovery of synaptic density at the hippocampus ( Fig. 7 A and B). This was further confirmed by analysing spine density in CA1 neurons by Golgi-Cox staining ( Fig. 7 C and D). We observed that treatment with GW2580 caused a significant prevention of the loss of dendritic spines observed in APP/PS1 mice ( Fig. 7 C and D).

The observed beneficial effects of prolonged and specific targeting of CSF1R, with the orally available inhibitor GW2580, provide a proof of target engagement and efficacy in a model of Alzheimer’s disease-like pathology.

## Discussion

The innate immune component has a clear influence over the onset and progression of Alzheimer’s disease. The analysis of therapeutic approaches aimed at controlling neuroinflammation in Alzheimer’s disease is moving forward at the preclinical and clinical level, with several clinical trials aimed at modulating inflammatory components of the disease. We have previously demonstrated that the proliferation of microglial cells is a core component of the neuroinflammatory response in a model of prion disease, another chronic neurodegenerative disease, and is controlled by the activation of CSF1R ( Gomez-Nicola *et al.* , 2013 ). This aligns with recent reports pinpointing the causative effect of the activation of the microglial proliferative response on the neurodegenerative events of human and mouse Alzheimer’s disease, highlighting the activity of the master regulator PU.1 ( Gjoneska *et al.* , 2015 ). Our results provide a proof of efficacy of CSF1R inhibition for the blockade of microglial proliferation in a model of Alzheimer’s disease-like pathology. Treatment with the orally available CSF1R kinase-inhibitor (GW2580) proves to be an effective disease-modifying approach, partially improving memory and behavioural performance, and preventing synaptic degeneration. These results support the previously reported link of the inflammatory response generated by microglia in models of Alzheimer’s disease with the observed synaptic and behavioural deficits, regardless of amyloid deposition ( Jones and Lynch, 2014 ). Our findings support the relevance of CSF1R signalling and microglial proliferation in chronic neurodegeneration and validate the evaluation of CSF1R inhibitors in clinical trials for Alzheimer’s disease.

Our findings show that the inhibition of microglial proliferation in a model of Alzheimer’s disease-like pathology does not modify the burden of amyloid-β plaques, suggesting an uncoupling of the amyloidogenic process from the pathological progression of the disease. Although a recent report correlated the accumulation of microglia with the expansion of amyloid-β plaques, suggesting that microglial cells could act as a ‘barrier’ ( Condello *et al.* , 2015 ), our current data provide evidence that removing microglia does not alter the deposition of amyloid-β. This indicates a necessity of the executive role of microglia to linking amyloid deposition with the progression of cognitive decline. In this direction, recent reports using *Trem2*^+/−^ mice on an APP/PS1 background evidenced an impaired amplification of the microglia population in response to amyloid-β, suggesting that deficient phagocytosis could link with impaired microglial proliferation ( Ulrich *et al.* , 2014 ). Linking TREM2 activation with CSF1R-induced proliferation could be possible through the adaptor molecule DAP12, as suggested for macrophages ( Otero *et al.* , 2009 ), although the functional connection of amyloid-β phagocytosis and microglial proliferation has not been reported to date. Our results are in agreement with previously reported data using CD11b-HSVTK mice under two different APP models, in which microglial cells were depleted after treatment with ganciclovir ( Grathwohl *et al.* , 2009 ). The authors found no change in the number of amyloid-β plaques despite the fact that the brains of APP or APP/PS1 mice were virtually devoid of microglia. Although the amyloid-β accumulation is still considered a main driver of the pathogenic events in Alzheimer’s disease, several studies support the notion that additional factors are necessary for the development of the cognitive decline. Amyloid-β and tau accumulation are frequently observed in the brains of non-demented people ( Neuropathology Group; Medical Research Council Cognitive and Aging, 2001 ). Also, patients immunized against amyloid-β, leading to the effective removal of their plaques, continued to decline in cognitive function ( Boche *et al.* , 2008 ). Evidence suggests that inflammation ( Holmes *et al.* , 2009 ), tangle pathology ( Nelson *et al.* , 2012 ) or neurodegeneration-related biomarkers ( Wirth *et al.* , 2013 ) correlate better with cognitive decline than amyloid-β accumulation alone. All this evidence, together with our present data, support an uncoupling of some pathological hallmarks of Alzheimer’s disease from amyloid-β, and highlight a more indirect route through the inflammatory component. A better understanding of what processes are amyloid-β and/or tau dependent and which ones are caused, or exacerbated by inflammation, would be a way forward to dissect the pathophysiology of Alzheimer’s disease.

Interestingly, the report by Grathwohl *et al.* (2009) indirectly supports our present findings regarding a relatively high rate of microglial proliferation in APP/PS1 mice. The fast depletion of microglia in CD11b-HSVTK (2 or 4-weeks with ganciclovir) would only occur if all microglia were entering the cell cycle during that time, as thymidine kinase can only kill dividing cells. Although this proliferation rate is high it does suggest that other factors, such as the recently reported anti-proliferative actions of ganciclovir alone ( Ding *et al.* , 2014 ), are contributing to the observed decline in microglia in CD11b-HSVTK mice ( Grathwohl *et al.* , 2009 ). Our data suggest that, on average in 9- and 14-month-old APP/PS1 mice, the proliferation index (PI = Iba1 ^+^ BrdU ^+^ / total Iba1 ^+^ ) is 1.9%, suggesting that an estimated 53% of the microglia undergo proliferation during a 4-week period. This high rate is similar with that found in human samples, where the proliferation index (PI = Iba1 ^+^ Ki67 ^+^ / total Iba1 ^+^ ) in Alzheimer’s disease brains is 2.63% in grey matter and 1.52% in white matter. The fact that the mild increase in total microglial numbers in both APP/PS1 and human Alzheimer’s disease is not justified by the reported high rates of proliferation indicates the necessity of compensatory microglial death. This aspect needs further exploration in future work, to better understand the observed discrepancies in different brain regions (i.e. human cortical grey versus white matter). It is worth noting that the expansion of the microglial population in the model used in the current study, APP/PS1, courses without the contribution of circulating monocytes, as evidenced by the use of CCR2 deficient mice and bone-marrow transplant with head shielding ( Mildner *et al.* , 2011 ).

While the mechanistic link of CSF1R with the observed beneficial outcomes seems clear, there are variables to take into account when evaluating potential candidate drugs targeting CSF1R. The activation of CSF1R can be achieved by two independent mitogens, CSF1 and IL34 ( Hume and MacDonald, 2012 ). Although both CSF1 and IL34 are expressed in many organs, IL34 appears particularly upregulated in the developing and adult brain, suggesting specific functions that do not overlap with those of CSF1 ( Wei *et al.* , 2010 ). Recently, data arising from the generation of IL34 ^LacZ/LacZ^ mice showed that IL34 is a tissue-restricted ligand, controlling the development of Langerhans cells in the skin and microglia in the brain ( Wang *et al.* , 2012 ). In contrast, targeted deletion of IL34 had little effect on other myeloid-cell compartments and dendritic cell subsets were largely unaffected ( Greter *et al.* , 2012 ; Wang *et al.* , 2012 ). In conclusion, these studies suggest that the maintenance of the populations of microglia and Langerhans cells is dependent on IL34–CSF1R signalling, supporting the particular ability of these cell populations to self-renew throughout life ( Merad *et al.* , 2008 ; Ginhoux *et al.* , 2013 ). Therefore, and although our present data do not show any significant change in IL34 levels, it would be interesting to observe the impact of anti-IL34 therapeutic approaches to provide a more targeted inhibition of the functions controlled by CSF1R in the brain, without affecting many populations in peripheral organs. This is a particularly relevant issue to be addressed, as a prolonged systemic delivery of CSF1R inhibitors may cause an unbalanced response in CSF1R-dependent cells ( Sauter *et al.* , 2014 ). The therapeutic potential of CSF1R inhibitors has been suggested in inflammatory diseases, autoimmune disorders, bone disease and cancer ( Burns and Wilks, 2011 ; Pyonteck *et al.* , 2013 ). Monocyte-derived cell types are most likely required for the long-term integrity of the immune system. Therefore, the therapeutic benefit of influencing their function in particular pathologies may result in the alteration of the natural balance of the immune system, the consequences of which are hard to predict. In this direction, the use of imatinib (a PDGFR inhibitor with potent activity over CSF1R) in clinical trials has shown that these fears are unfounded, not presenting immune-related side effects ( Burns and Wilks, 2011 ). Similarly, an orally available inhibitor, JNJ-28312141, has substantial activity against CSF1R and the related receptor FLT3 ( Manthey *et al.* , 2009 ). JNJ-28312141 was found to decrease Kupffer cell numbers by 40%, and also to reduce macrophage numbers in transplanted tumours, constraining tumour growth ( Manthey *et al.* , 2009 ). Therefore, a more targeted analysis of the peripheral effects of CSF1R inhibition in ongoing or planned clinical trials is necessary, to better ponder the long-term side effects of potential therapies for Alzheimer’s disease.

One issue that requires comment is the selectivity of CSF1R inhibitor GW2580. CSF1R belongs to the family of type III growth factor receptors, including PDGFR, c-KIT, FLT3 and c-fms (CSF1R). The structural similarity of these receptors favours CSF1R inhibitors having certain degree of promiscuity, potentially influencing other targets ( Hume and MacDonald, 2012 ). An analysis of the activity and selectivity of several CSF1R inhibitors confirmed the high selectivity of GW2580 for CSF1R, as all the other compounds studied were also inhibitors of PDGFRβ and c-Kit ( Uitdehaag *et al.* , 2011 ). A novel inhibitor, PLX3397, has been described to have potent activity over CSF1R, inhibiting the survival of microglia in the healthy brain and causing the rapid depletion of the population ( Elmore *et al.* , 2014 ). However, PLX3397 also has potent inhibitory activity over c-Kit, FTL3 and PDGFRβ ( Patwardhan *et al.* , 2014 ), confounding the reported effects on the microglial population. Loss of PDGFβ signalling, for example, would be expected to have an impact on survival of the NG2 pericytes leading to damage of the blood–brain barrier and neurodegeneration ( Bell *et al.* , 2010 ). The particular effects of these approaches on the microglial population highlight an important aspect: a therapeutic approach to control CSF1R activity should be effective in inhibiting microglial proliferation, but not affecting the survival of the remaining microglial cells, compromising otherwise the normal brain function ( Gomez-Nicola and Perry, 2014 *a* , *b* ). Our evidence shows that GW2580 selectively inhibits microglial proliferation, and not survival, as long-term dosing does not cause a significant reduction in the number of microglial cells in wild-type mice. CSF1R activation has pleiotropic effects, ranging from the control of cell survival, proliferation or chemotaxis, based on the differential binding of adapter proteins and phosphorylation pattern of its intracellular domain ( Pixley and Stanley, 2004 ). Evidence from conformational analysis supports the idea that different CSF1R inhibitors can bind the active or inactive forms of the receptor and also show different dissociation rates, suggesting that these biophysical properties could underpin a differential functional activation of CSF1R ( Uitdehaag *et al.* , 2011 ). These ideas need to be taken into account to fully understand the effects of a sustained inhibition of CSF1R in the normal physiology of the brain.

In summary, the present data provide strong evidence for an increased proliferative response in microglia in Alzheimer’s disease, as well as their dependence upon CSF1R activation. Prolonged reduction of microglial activation and proliferation in Alzheimer’s disease mice using a selective CSF1R inhibitor prevents cognitive decline, regardless of amyloid plaque pathology. We provide support for the efficacy of CSF1R inhibitory strategies in the treatment of Alzheimer’s disease-like pathology to reduce microglia numbers and reduce the potentially damaging components of neuroinflammation, thus underpinning the possible evaluation of CSF1R inhibitors in clinical trials for Alzheimer’s disease.

## Supplementary Material

Supplementary Data

Supplementary Table 1

## Abbreviation

BrdU: bromodeoxyuridine
