# Supplementary material for: Pharmacological targeting of CSF1R inhibits microglial proliferation and prevents the progression of Alzheimer’s-like pathology
Source: Brain. 2016 Jan 8;139(3):891–907. doi: 10.1093/brain/awv379 (PMC4766375; doi:10.1093/brain/awv379)
Supplement: Supplementary Data [file awv379_supplementary_data.zip › brain-2015-01261-File012.pdf]

DIAGNOSIS	AGE	SEX	PM DELAY	CAUSE OF DEATH	SI	AGE ONSET	DURATION	BRAAK STAGE	APOE
NDC	64	M	16	Ruptured atherosclerotic abdominal aortic aneurysm	N	-	-	0	3/3
NDC	68	F	38.75	Pulmonary hypertension & multiple pulmonary emboli	N	-	-	0	2/3
NDC	72	F	24	Unknown	N	-	-	0	3/3
NDC	80	M	45.75	Pneumonia	Y	-	-	0	3/3
NDC	86	F	32	Caecal carcinoma of colon	N	-	-	2	3/4
NDC	87	F	47	Septicaemia, uti, broncial pneumonia	Y	-	-	3	2/3
NDC	89	M	91	Bronchopneumonia, chronic obstructive airways disease	Y	-	-	2	3/4
AD	60	F	18.75	Aspiration pneumonia	Y	54	6	6	3/3
AD	64	M	66.5	Alzheimer's dementia	N	55	10	5	4/4
AD	80	M	24	Bronchopneumonia	Y	77	3	4	3/4
AD	80	F	26	Gist tumour	N	73	7	4	3/4
AD	83	M	11	Dementia	N	81	2	5	3/3
AD	84	F	22	Alzheimer's disease	N	80	4	5	ND
AD	86	F	45.25	Urinary tract sepsis	Y	75	12	6	ND
AD	88	M	28	Bronchial pneumonia	Y	85	3	6	ND
