# Supplementary material for: Pharmacological targeting of CSF1R inhibits microglial proliferation and prevents the progression of Alzheimer’s-like pathology
Source: Brain. 2016 Jan 8;139(3):891–907. doi: 10.1093/brain/awv379 (PMC4766375; doi:10.1093/brain/awv379)
Supplement: Supplementary Data [file awv379_supplementary_data.zip › brain-2015-01261-File013.pdf]

	Common name	Gene name	Taqman probe	Braak Correlation (R^2)
**Microglia/ Macrophage markers**	CD11b	ITGAM	Hs00355885_m1	0.440*
	CD11c	ITGAX	Hs00174217_m1	0.419*
	CD163	CD163	Hs00174705_m1	0.501*
	CD68	CD68	Hs02836816_g1	0.440*
	CX3CR1	CX3CR1	Hs01922583_s1	0.501*
	IBA1	AIF1	Hs00610419_g1	0.297
**HSCs/BMDCs markers**	C-KIT	KIT	Hs00174029_m1	-0.031
	C-MYB	MYB	Hs00920556_m1	-0.133
	CCL2, MCP1	CCL2	Hs00234140_m1	0.563**
	CCR2	CCR2	Hs00704702_s1	0.481*
	CD34	CD34	Hs00990732_m1	0.522**
	Ly6C	CD59	Hs00174141_m1	0.473*
	SCA1	ATXN1	Hs00165656_m1	-0.256
**Microglial/Cell proliferation markers**	C/EBPa	CEBPA	Hs00269972_s1	0.576**
	CSF1	CSF1	Hs00174164_m1	0.583**
	CSF1R	CSF1R	Hs00911250_m1	0.379
	Cyclin D1	CCND1	Hs00765553_m1	0.349
	Cyclin D2	CCND2	Hs00153380_m1	0.051
	IL34	IL34	Hs00380956_m1	-0.010
	Ki67	MKI67	Hs01032443_m1	0.072
	PCNA	PCNA	Hs00427214_g1	0.604**
	PU.1	SPI1	Hs02786711_m1	0.481*
	RUNX1	RUNX1	Hs01021971_m1	0.338
**Inflammation**	C/EBPb	CEBPB	Hs00270923_s1	0.440*
	CX3CL1	CX3CL1	Hs00171086_m1	-0.419*
	IGF1	IGF1	Hs01547656_m1	-0.256
	IL10	IL10	Hs00961622_m1	0.235
	IL1b	IL1B	Hs01555410_m1	0.072
	IL6	IL6	Hs00985639_m1	0.215
	IRF8	IRF8	Hs00175238_m1	0.358
	TGFb	TGFB1	Hs00998133_m1	0.522**
**Other**	Cystatin F	CST7	Hs00175361_m1	0.747**
	Dectin 1	CLEC7A	Hs01902549_s1	0.481*
	GPNMB	GPNMB	Hs01095679_m1	0.460*
	LILRB4	LILRB4	Hs00429000_m1	0.379
	Osteopontin	SPP1	Hs00959010_m1	0.726**
	TREM2	TREM2	Hs00219132_m1	0.522**
	TYROBP	TYROBP	Hs00182426_m1	0.501*
