# Supplementary material for: Pharmacological targeting of CSF1R inhibits microglial proliferation and prevents the progression of Alzheimer’s-like pathology
Source: Brain. 2016 Jan 8;139(3):891–907. doi: 10.1093/brain/awv379 (PMC4766375; doi:10.1093/brain/awv379)
Supplement: Supplementary Data [file awv379_supplementary_data.zip › brain-2015-01261-File016.pdf]

Supplementary Figure 1. Distribution of CSF1R$^+$ microglia in APP/PS1 mice.

Immunofluorescent analysis of the expression of CSF1R (green) around A□ plaques (6E10, red) in the cortex of APP/PS1 mice at 14 months of age. Nuclei are shown in blue (DAPI).

Supplementary Figure 2. Expression of CSF1R in APP/PS1 mice.

Confocal analysis of the expression of CSF1R in microglia, detected by the expression of EGFP under the c-fms promoter (APP/PS1/macgreen mice). Note lack of expression in neurons (NeuN$^+$, red) or astrocytes (GFAP$^+$, blue).

Supplementary Figure 3. Effect of GW2580 on the survival of microglial cells.

(A) Immunohistochemical analysis and quantification of the number of total microglial cells (Iba1$^+$) in the cortex and hippocampus of WT mice, after treatment for 5 days with GW2580 by oral gavage (75, 150 and 300 mg/kg). (**B**) Number of microglia represented as mean±SEM of Iba1$^+$ cells/mm^2.

Table S1. Pathology of the selected post-mortem cases of AD and age-matched controls.

Pathology of the post-mortem samples from AD or age-matched non-demented controls (NDC) used in this study. PM Delay, post-mortem delay (h); SI, presence of concomitant systemic inflammatory event at death, according to cause of death; ApoE, ApoE genotype. Data provided by the South-West Dementia Brain Bank (SWDBB).

Table S2. List of genes analysed by RT-PCR in human AD tissue and correlation of gene expression with Braak score.

List of the genes (name and Taqman probe ID) analysed in this study in post-mortem samples

from AD or age-matched non-demented controls (NDC). Data arising from the correlation of gene expression with the Braak score is shown in red for comparisons showing significant correlation in the Kendall tau-b rank correlation test. Correlation coefficient (R^2) is shown, together with statistical differences as $^{*}p<0.05$, $^{**}p<0.01$.
