# Supplementary figures and images for: Pharmacological targeting of CSF1R inhibits microglial proliferation and prevents the progression of Alzheimer’s-like pathology
Source: Brain. 2016 Jan 8;139(3):891–907. doi: 10.1093/brain/awv379 (PMC4766375; doi:10.1093/brain/awv379)

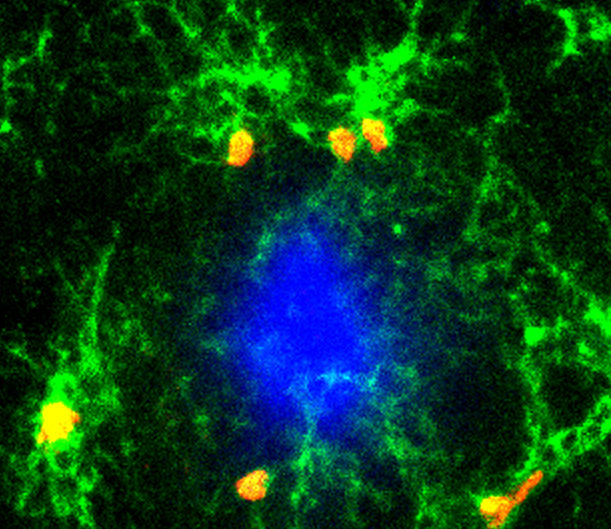

Supplement: Supplementary Data [file awv379_supplementary_data.zip › brain-2015-01261-File014.tif]

CSF1R

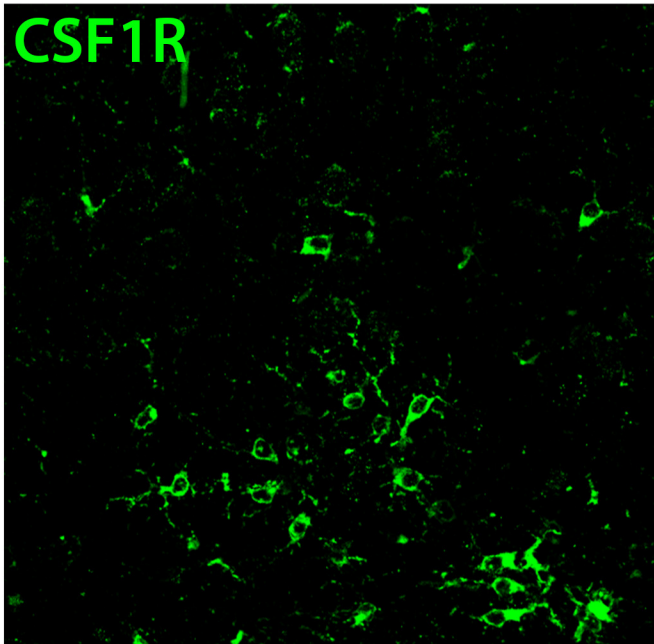

A $\beta$

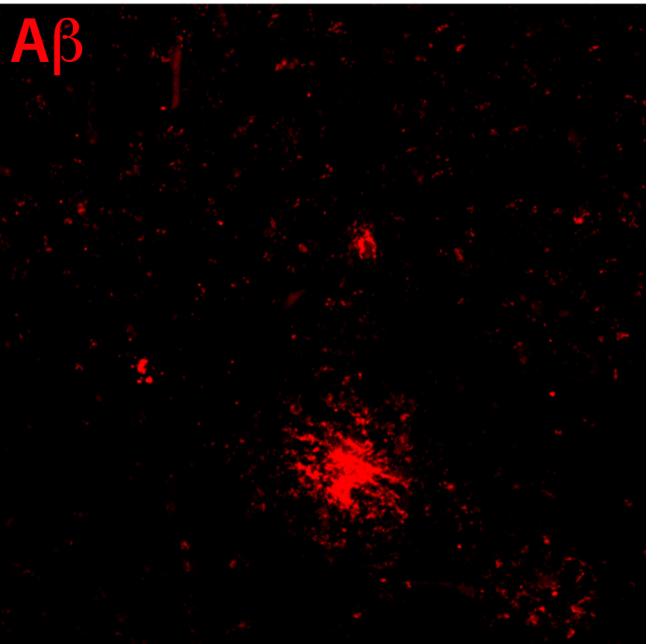

CSF1R/A $\beta$

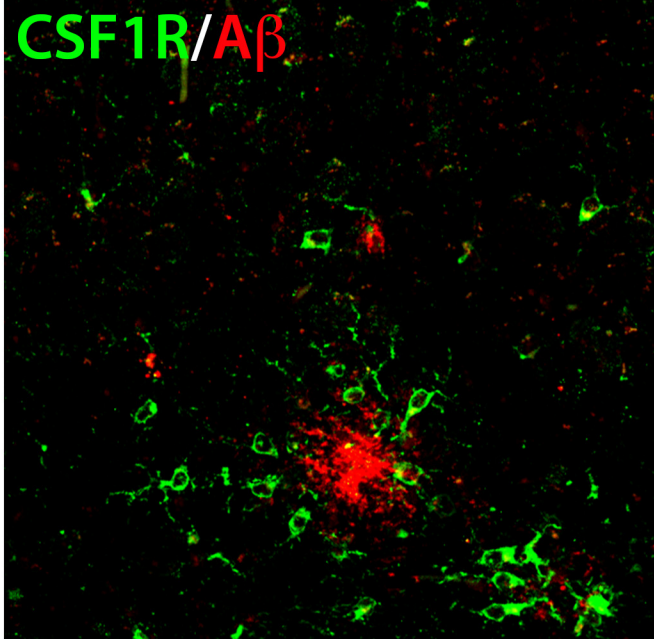

CSF1R/A $\beta$ /DAPI

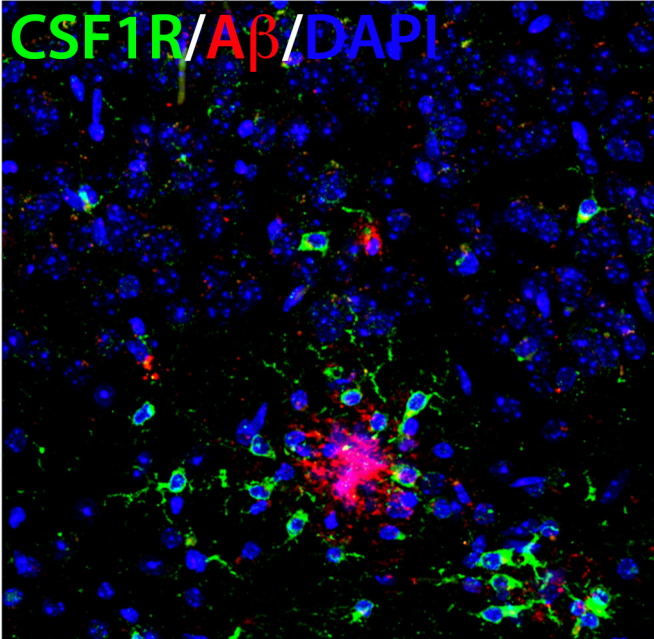

Supplement: Supplementary Data [file awv379_supplementary_data.zip › brain-2015-01261-File009.pdf]

APPPS1 9m

c-fms EGFP/NeuN

APPPS1 9m

c-fms EGFP/NeuN/GFAP

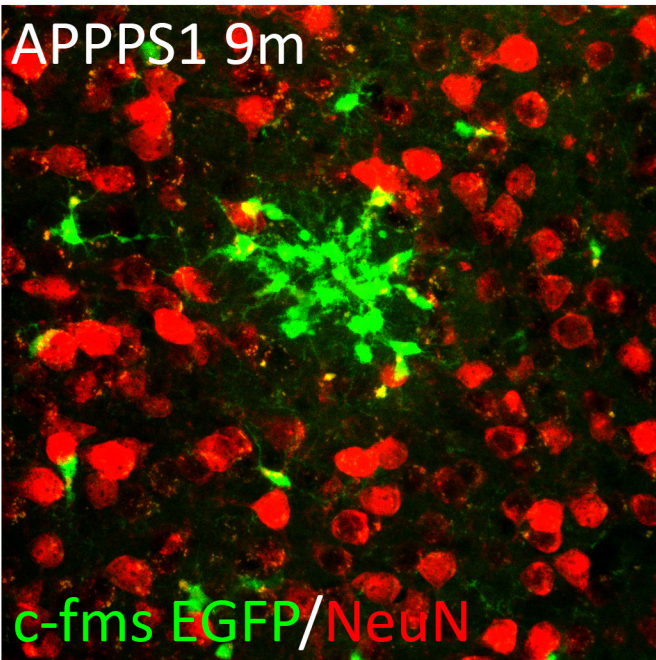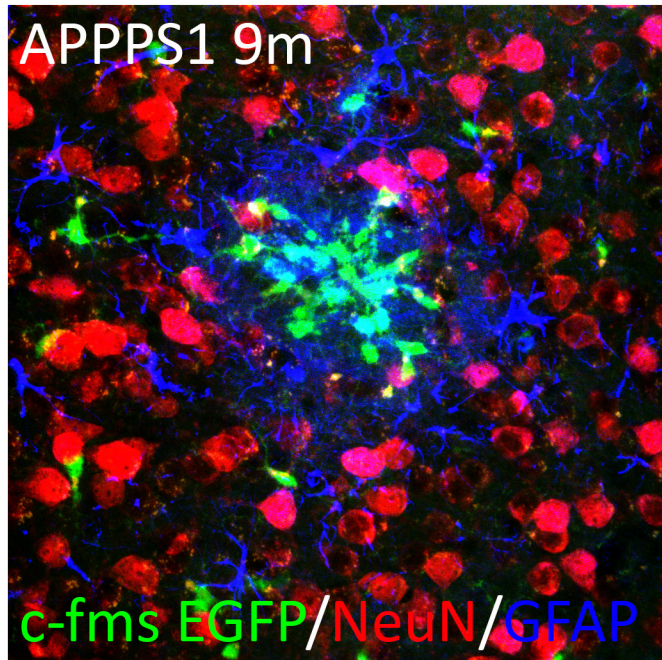

Supplement: Supplementary Data [file awv379_supplementary_data.zip › brain-2015-01261-File010.pdf]

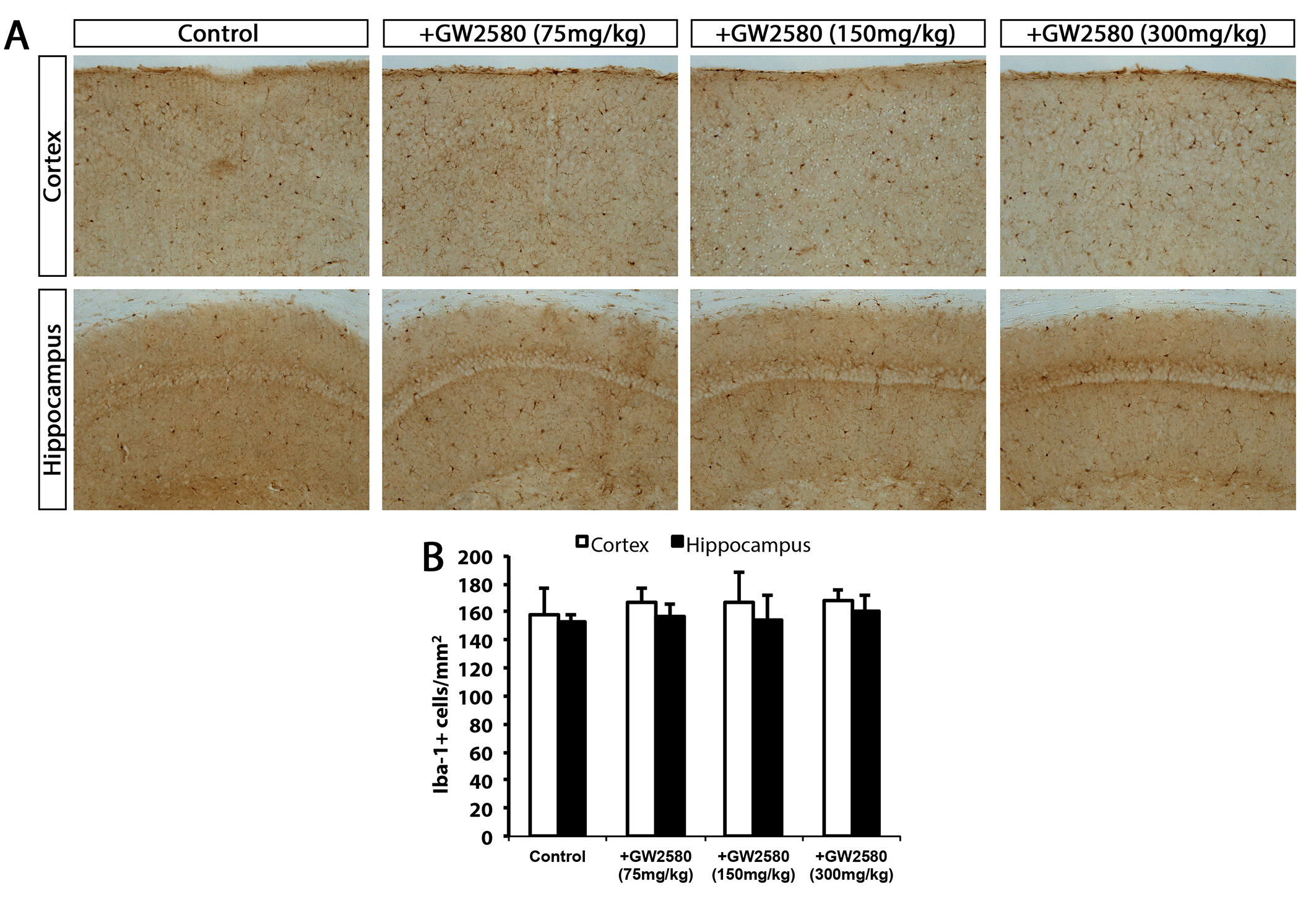

Supplement: Supplementary Data [file awv379_supplementary_data.zip › brain-2015-01261-File011.jpg]

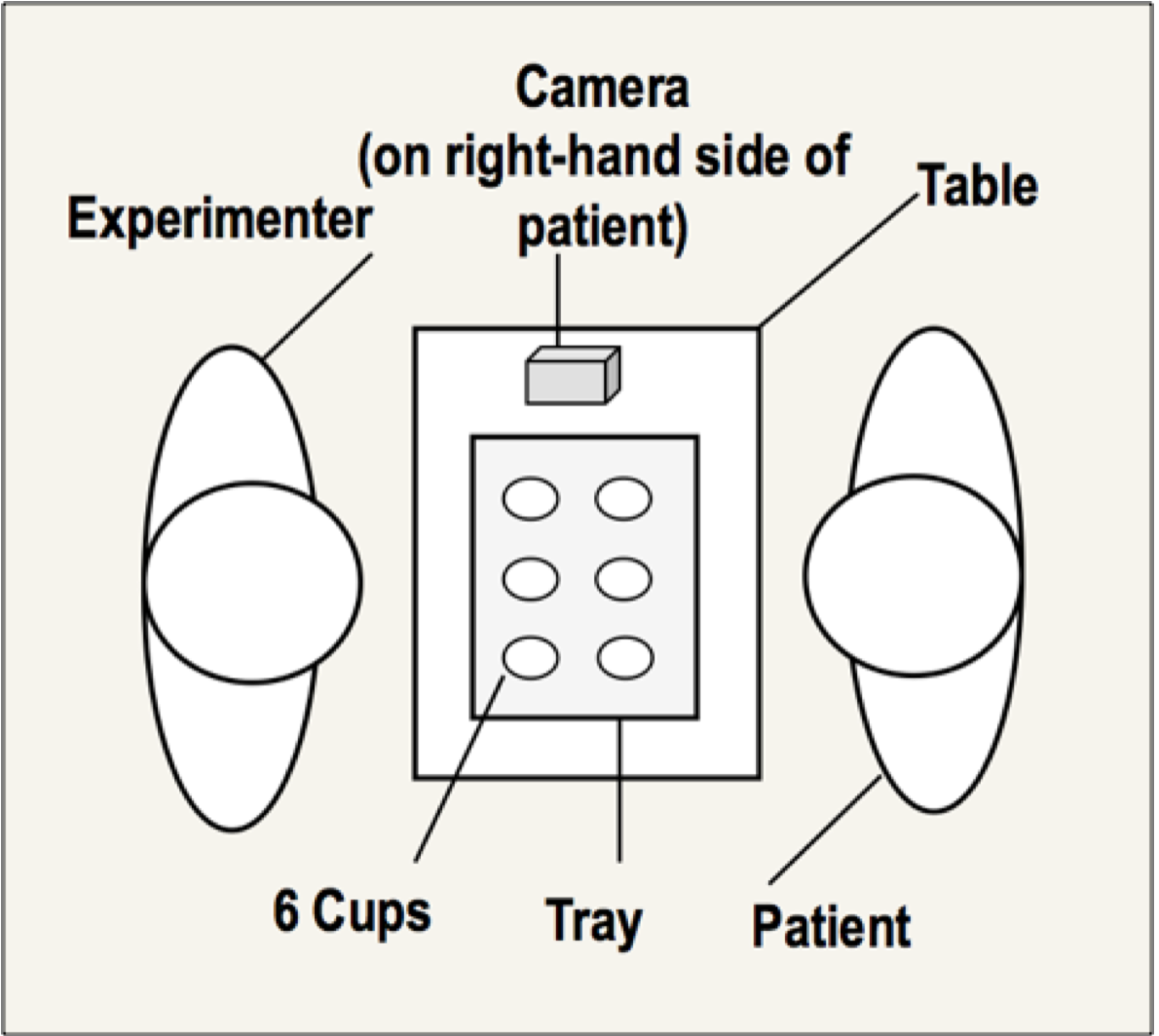

Supplement: Supplementary Table 1 [file suppl_data.zip › brain-2015-00799-File015.tiff]
