# Supplementary material for: Pharmacological targeting of CSF1R inhibits microglial proliferation and prevents the progression of Alzheimer’s-like pathology
Source: Brain. 2016 Jan 8;139(3):891–907. doi: 10.1093/brain/awv379 (PMC4766375; doi:10.1093/brain/awv379)
Supplement: Supplementary Table 1 [file suppl_data.zip › brain-2015-00799-File011.pdf]

Supplementary Table 2. *Comparison of anosognosic patients with hemiplegic control patients on the visual spatial perspective taking task (using Crawford, Garthwaite & Porter (2010) Revised Standardised Difference Test).*

	1st person perspective[a]			3rd person perspective[b]			Dissociation Test[c]	
**Patient**	**Score (% correct)**	***t***	***p* (1-tailed)**	**Score (% correct)**	***t***	***p* *(1-tailed)***	***t***	***p* *(1-tailed)***
AHP								
1/RK	100	0.25	.403	25	-1.31	.106	1.17	.131
2/GU	100	0.25	.403	25	-1.31	.106	1.17	.131
3/CA	100	0.25	.403	50	-0.52	.305	0.58	.285
4/AB	100	0.25	.403	0	-2.09	.028*	1.76	.051
5/GA	50	-3.50	.002*	25	-1.31	.106	1.65	.061
6/JT	100	0.25	.403	25	-1.31	.106	1.17	.131
7/JM	100	0.25	.403	50	-0.52	.305	0.58	.285
8/CD	100	0.25	.403	0	-2.09	.028*	1.76	.051
9/OL	50	-3.50	.002*	0	-2.09	.028*	1.06	.154
10/MM	100	0.25	.403	0	-2.09	.028*	1.76	.051
11/MO	100	0.25	.403	0	-2.09	.028*	1.76	.051
12/CP	100	0.25	.403	0	-2.09	.028*	1.76	.051
13/GK	100	0.25	.403	0	-2.09	.028*	1.76	.051
14/SA	100	0.25	.403	50	-0.52	.305	0.58	.285
15/IB	100	0.25	.403	0	-2.09	.028*	1.76	.051

[a] Hemiplegic control mean = 96.67; SD = 12.91; N = 15.
[b] Hemiplegic Control mean = 66.67; SD = 30.86; N = 15.
[c] Correlation between 1st and 3rd person tasks in hemiplegic control sample = .193.
* significant deficit.
