# Supplementary material for: Pharmacological targeting of CSF1R inhibits microglial proliferation and prevents the progression of Alzheimer’s-like pathology
Source: Brain. 2016 Jan 8;139(3):891–907. doi: 10.1093/brain/awv379 (PMC4766375; doi:10.1093/brain/awv379)
Supplement: Supplementary Table 1 [file suppl_data.zip › brain-2015-00799-File012.pdf]

Supplementary Table 3. *Comparison of anosognosic and hemiplegic control patients with healthy controls on the Theory of Mind task (using Crawford, Garthwaite & Porter (2010) Revised Standardised Difference Test).*

	1st person perspective[a]			3rd person perspective[b]			Dissociation Test[c]	
**Patient**	**Score (% correct)**	***t***	***p* (1-tailed)**	**Score (% correct)**	***t***	***p (1-tailed)***	***t***	***p (1-tailed)***
AHP								
1/RK	87.5	0.15	.441	25	-3.15	.004*	4.84	<.001**
2/GU	81.25	-0.45	.329	31.25	-2.80	.007*	3.51	.002**
3/CA	93.75	0.76	.231	50	-1.77	.050*	3.76	.001**
4/AB	46.88	-3.78	.001*	25	-3.15	.004*	0.96	.177
5/GA	71.88	-1.36	.098	40	-2.32	.018*	1.46	.083
6/JT	90.63	0.45	.329	15.63	-3.67	.001*	5.93	<.001**
7/JM	96.88	1.06	.154	15.63	-3.67	.001*	6.70	<.001**
8/CD	87.5	0.15	.441	37.5	-2.46	.014*	3.88	.001**
9/OL	81.25	-0.45	.329	37.5	-2.46	.014*	3.01	.005**
10/MM	46.88	-3.78	.001*	6.25	-4.19	<.001*	0.63	.271
11/MO	75	-1.06	.154	21.88	-3.32	.003*	3.39	.002**
12/CP	78.13	-0.76	.231	25	-3.15	.004*	3.58	.002**
13/GK	62.5	-2.27	.020*	46.88	-1.94	.037*	0.50	.311
14/SA	87.5	0.15	.441	50	-1.77	.050*	2.89	.006**
15/IB	50	-3.59	.002*	37.5	-2.46	.014*	1.55	.072
HP Controls								
1/RS	93.75	0.76	.231	75	-0.38	.355	1.73	.053
2/BD	50	-3.48	.002*	68.75	-0.73	.240	4.08	.001
3/NBD	81.63	-0.42	.342	69.12	-0.71	.246	0.44	.333
4/DS	100	1.36	.098	75	-0.38	.355	2.63	.010
5/BSG	78.5	-0.72	.242	72.25	-0.53	.301	0.29	.390
6/ND	56.25	-2.87	.006*	59.38	-1.25	.117	2.46	.014
7/SC	62.5	-2.27	.020*	43.75	-2.11	.027*	0.24	.407
8/DM	46.88	-3.78	.001*	65.63	-0.9	.192	4.26	.001
9/AM	81.25	-0.45	.329	90.63	0.48	.318	1.43	.088
10/FB	87.5	0.15	.441	87.5	0.31	.380	0.25	.405
11/PR	87.5	0.15	.441	56.25	-1.42	.089	2.38	.016
12/IJ	68.75	-1.66	.059	81.25	-0.04	.486	2.46	.014
13/SM	87.5	0.15	.441	68.75	-0.73	.240	1.34	.101
14/KR	75.38	-1.02	.162	62.87	-1.05	.155	0.05	.482
15/JR	75	-1.06	.154	56.25	-1.42	.089	0.55	.295

[a] Healthy Control mean = 85.94; SD = 10.01 N = 15.
[b] Healthy Control mean = 81.88; SD = 17.49; N = 15.
[c] Correlation between 1st and 3rd person tasks in healthy control sample = .813.
* significant deficit
** significant dissociation (differential deficit) between unimpaired first person perspective taking ability and third person perspective taking deficit.
