# Supplementary material for: Pharmacological targeting of CSF1R inhibits microglial proliferation and prevents the progression of Alzheimer’s-like pathology
Source: Brain. 2016 Jan 8;139(3):891–907. doi: 10.1093/brain/awv379 (PMC4766375; doi:10.1093/brain/awv379)
Supplement: Supplementary Table 1 [file suppl_data.zip › brain-2015-00799-File013.pdf]

Supplementary Table 4. *Comparison of anosognosic patients with hemiplegic control patients on the Theory of Mind task (using Crawford, Garthwaite & Porter (2010) Revised Standardised Difference Test).*

	1st person perspective[a]			3rd person perspective[b]			Dissociation Test[c]	
**Patient**	**Score (% correct)**	***t***	***p* (1-tailed)**	**Score (% correct)**	***t***	***p (1-tailed)***	***t***	***p (1-tailed)***
AHP								
1/RK	87.5	0.74	.237	25	-3.43	.002*	3.63	.002**
2/GU	81.25	0.35	.365	31.25	-2.94	.005*	2.89	.006**
3/CA	93.75	1.12	.141	50	-1.47	.081	2.28	.017
4/AB	46.88	-1.75	.051	25	-3.43	.002*	1.48	.081
5/GA	71.88	-0.22	.414	40	-2.26	.020*	1.79	.048**
6/JT	90.63	0.93	.185	15.63	-4.16	<.001*	4.41	.001**
7/JM	96.88	1.31	.106	15.63	-4.16	<.001*	4.72	<.001**
8/CD	87.5	0.74	.237	37.5	-2.45	.014*	2.80	.007**
9/OL	81.25	0.35	.365	37.5	-2.45	.014*	2.46	.014**
10/MM	46.88	-1.75	.051	6.25	-4.89	<.001*	2.76	.008**
11/MO	75	-0.03	.488	21.88	-3.67	.001*	3.19	.004**
12/CP	78.13	0.16	.436	25	-3.43	.002*	3.14	.004**
13/GK	62.5	-0.80	.220	46.88	-1.72	.054	0.82	.215
14/SA	87.5	0.74	.237	50	-1.47	.081	1.95	.036
15/IB	50	-1.56	.070	37.5	-2.45	.014*	0.79	.222

[a] Hemiplegic control mean = 75.49; SD = 15.81; N = 15.
[b] Hemiplegic Control mean = 68.83; SD = 12.38; N = 15.
[c] Correlation between 1st and 3rd person tasks in hemiplegic control sample = .426.
* significant deficit.
** significant dissociation (differential deficit) between unimpaired first person perspective taking ability and third person perspective taking deficit.
