# Supplementary material for: Pharmacological targeting of CSF1R inhibits microglial proliferation and prevents the progression of Alzheimer’s-like pathology
Source: Brain. 2016 Jan 8;139(3):891–907. doi: 10.1093/brain/awv379 (PMC4766375; doi:10.1093/brain/awv379)
Supplement: Supplementary Table 1 [file suppl_data.zip › brain-2015-00799-File009.pdf]

Supplementary materials

1. Pilot results for visuospatial perspective taking task (VSPT) and Theory of Mind (ToM) stories

The VSPT task was piloted on 4 patients (2 AHP and 2 HP patients), testing for comprehension of questions and possible effects of visuospatial neglect. The position of the camera (inanimate other) and experimenter (other) was changed across various trials between positions (at a 90° and 180° angle). Results indicated that the position of the camera or experimenter made no difference in patient responses. The suitability and comprehension of experimental instructions and questions was also confirmed, as well as controlling for the influence of visuospatial neglect.

ToM stories were initially piloted on 20 healthy participants testing for readability and comprehension of stories, as well as correct multiple-choice responses. The results confirmed the suitability of the stories and questions, but minor corrections were made on the readability of the specific stories. Accordingly, three of the stories were adjusted (e.g. sentences shortened and vocabulary modified) according to the responses of the participants. ToM stories were further piloted on 4 patients (2 AHP and 2 HP patients), testing for: the comprehension and readability of stories; the length of the experimental protocol and engagement of patients; and their ability to make spontaneous answers and choose multiple-choice options. Four of the stories were modified as a result, with sentences being simplified for enhanced accessibility of the material. Patients were readily able to engage with the experimental material, with dividing the administration of story sets between 2 separate sessions, as well as repetition of stories and questions when requested, helping with fatigue and engagement. All patients were also able to make spontaneous answers and select multiple-choice options when verbally presented.

2. Examples of visual arrangement for visuospatial perspective taking (VSPT) task and Control questions and arrangements for visuospatial perspective-taking (VSPT) task

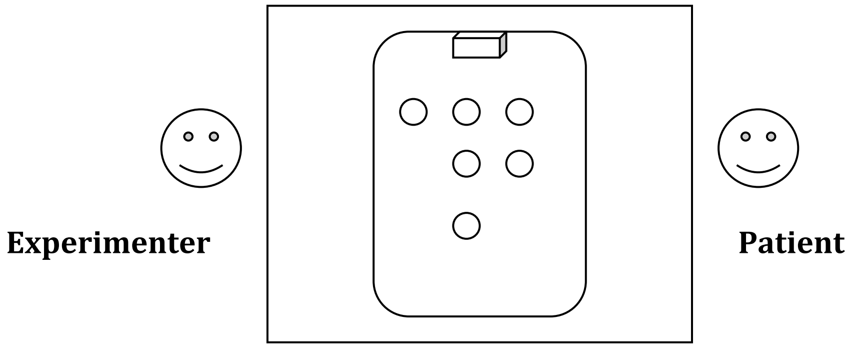

Example 1 of visual arrangements of position of cups on tray.

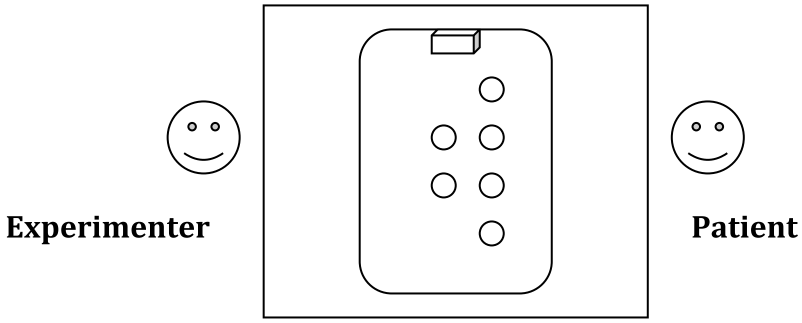

Example 2 of visual arrangements of position of cups on tray.

Say:

> "Now we are going to do something a little different. I am going to ask you a few questions involving this tray and cups. The questions will be about the position of the cups. I will ask you some questions about how you or I see the cups or about what pictures the camera might take. Do you understand?"

Then ask:

"Can you see the Camera*?",* if they cannot see the camera, try to move it to their right visual gaze.

Then focus their attention to the tray and cups, and ask, "Can you see the tray and cups?" Try and move the tray and cups until it is their full visual field.

Ask the following questions to confirm that the patient can clearly see the tray and cups: "How many cups do you see?", "What colour is the tray?"
Only continue after you have made sure the patient can see the tray and all the cups.

Set up the trays and cups as in the diagram below, again make sure the patient can see all the cups. The camera should be on the RIGHT side of the patient.

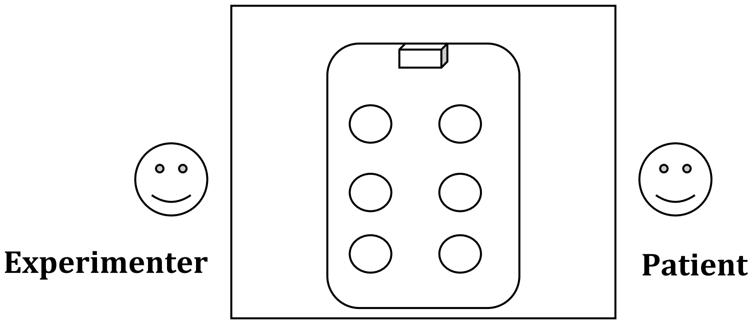

3. Full description of procedures for Theory of Mind (ToM) stories and examples of Theory of mind (ToM) stories from both 1st person perspective taking and 3rd person perspective-taking set

Procedures

All scenarios and questions were read aloud to the participants, in a slow pace and neutral tone. Stories and questions were repeated on request of the participant or if the examiner felt it was necessary due to distraction or fatigue. Participants were self-paced and were given ample time for responding. The participants were first required to make a spontaneous response, which the examiner wrote down in full. Subsequently, the experimenter read the multiple-choice options and participants had to indicate their choice verbally, which all patients were able to do. Of the multiple-choice options given, there was only one possible correct answer, the other options being either (i) the incorrect belief or (ii) irrelevant or incoherent with the story. For each question a composite score was calculated using both the multiple-choice answers and the spontaneous answer. Multiple-choice answers were scored as 1 = correct and 0 = incorrect. Spontaneous answers were scored as 1 = correct, 0.5 = partially correct/inadequate and 0 = incorrect. Two raters scored the spontaneous answers independently. Interclass correlation coefficient of 0.95 indicated a good agreement between raters. Divergent scores (<1% of stories) were discussed and jointly agreed on. Total scores were converted into percentages and used in the statistical analyses. As spontaneous answers may vary based

on several neuropsychological factors, supplementary statistical analysis were also run using multiple-choice answers only, showing the same pattern of results.

In the patient groups, testing was conducted in two successive sessions to avoid fatigue. The order of the presentation of the two sets (1st PPT and 3rd PPT) was counterbalanced. Each set began and ended with a control story. To check for comprehension, following each control story, all participants were asked to rate how well they understood the story. A 5-point Likert-type scale was used (i.e. “Using this scale from one to five, how well did you understand the story? One being the lowest score, where you understood very little, and 5 being the highest score, where you understood the whole story”). The scale was read aloud to participants and also presented visually as a vertical scale on an A4 sheet of paper (from one to five), positioned in the right visual field in order to minimise possible unilateral visual neglect effects in the patient groups. Participants were familiarised with the rating scale before the experiment.

	Set 1	Set 2
**Perspective**	*1st person ToM story*	*3rd person ToM story*
**Narrative**	Late one night you are leaving the supermarket. You always get the bus home because you are afraid that if you walk home in the dark someone may attack and rob you. When leaving, you see a small child, about to walk home alone. You approach the child and ask, "Would you like me to walk you home?"	Lisa is terrible at returning books. Lisa often loses the books she borrows. Paul takes his book collection very seriously and would be very unhappy to lose a book. Lisa asks to borrow a book from Paul's collection, Paul replies, "Oh that one, it is not very good!"
**1st order question**	Why do you say that?	Why does Paul say that?
**Multiple choice questions**	a) You believe the child may get robbed and attacked.<br>b) You are making a joke.<br>c) You want to rob the child.	a) Paul wants to discourage Lisa from borrowing the book.<br>b) Paul does not like to read.<br>c) Paul found the book terribly boring.
**Extended narrative**	The child has been warned by his parents not to talk or go anywhere with strangers, and tells you that he is fine on his own.	After hearing Paul's advice, Lisa thinks she now needs to choose another book.
**2nd order question**	Why does the child think you offered to walk him home?	Why does Lisa think she needs to choose another book?
**Multiple choice questions**	a) The child thinks you are being nice.<br>b) The child thinks you might hurt him.<br>c) The child thinks you are old and confused.	a) Lisa thinks the first book is too long.<br>b) Lisa thinks Paul suspects her of losing the book.<br>c) Lisa thinks Paul does not think the book is very good.

4. Full description of control tasks

False Belief tasks

Task 1 – Age-adapted "Smarties" Task

A modified version of the "Smarties" task (Gopnik and Astington, 1988) was used. In order to make the experiment more appropriate for the target sample (i.e. elderly adults instead of children) the "Smarties" box (a box of sweets popular in North America and the UK) was replaced with a cigarette box, which was considered to be both age appropriate and easily recognisable. During the "cigarette" false belief task, participants are first shown a clearly recognisable cigarette box, which (unbeknownst to the participants) contained coins instead of cigarettes. Participants are then asked a control question "What do you think is inside the box?" and are expected to respond by stating the expected contents of the box (i.e. cigarettes). Answering this question correctly was a prerequisite to continue the task and is not included in the experimental questions below. Subsequently, the box is opened and emptied in front of the participants revealing that the content is in fact coins, showing that their initial belief is false. The coins are then returned to the box and the participants are asked a false belief question: "If your friend comes to visit you now, what will he/she think is inside the box?". Participants are then asked two control questions: "What did you think was inside when I first showed you the box?" and " What do you think is inside the box now?". As in the original "Smarties" task, a score of 1 was given for each question answered correctly and a score of 0 for incorrect responses (maximum score = 3).

Task 2 – The 'Sally-Anne' Task

Baron-Cohen *et al.*'s (1985) "Sally-Anne" false belief task was used. The procedure involves the presentation of five illustrations that make up a cartoon strip (presented on the right-visual field controlling for visuospatial neglect), which are accompanied by the experimenter giving a verbal description of the depicted events. The illustrations are shown on an A4 page with verbal descriptions following each scenario to guide the participant through the narrative. In the first scene the characters Sally and Anne are introduced, as well as a basket and a box. In the subsequent scenes, Sally hides the marble in a basket and leaves the scene. The cartoon then shows that while Sally is gone, Anne shifts the marble to a box. In the last scene,

Sally returns. The participant is asked a belief question: “Where will Sally first look for her marble?” This is followed by two control questions: one reality control question, “Where is the marble now?” and one memory control question, “Where did Sally put the marble in the beginning?”. As in the original protocol (Baron-Cohen et al., 1985) a score of 1 was given for each question answered correctly and a score of 0 for incorrect responses (maximum score = 3).

Mental rotation task

A mental rotation task (Vandenberg and Kuse, 1978; Neuburger *et al.*, 2011) was added as an additional control task to assess whether deficits in visuospatial perspective could be attributed to impairments in mental rotation ability. This was tested on a subset of patients (six AHP and HP patients, respectively). The mental rotation task involves two conditions: a letter condition where a picture of the letter “F” is used and an animal condition where a line drawing of an elephant is used. The patient is required to mentally rotate the stimuli in each condition. In each condition, the target stimulus is shown on the right-hand side and four comparison stimuli are also presented vertically on the right side. The comparison stimuli are presented using two of five possible rotation angles across both conditions: 90°, 135°, 225°, 270° and 315°. Two of the four comparison stimuli are correct (i.e. actual rotations of the target image) and two incorrect (left-right reversed mirror images of the target, rather than an angular rotation). The patient is asked to cross out the two correct comparisons. The order of presentation of the two conditions was counterbalanced.

5. Figure of 3rd animate and 3rd inanimate conditions in visuospatial perspective taking (VSPT) tasks

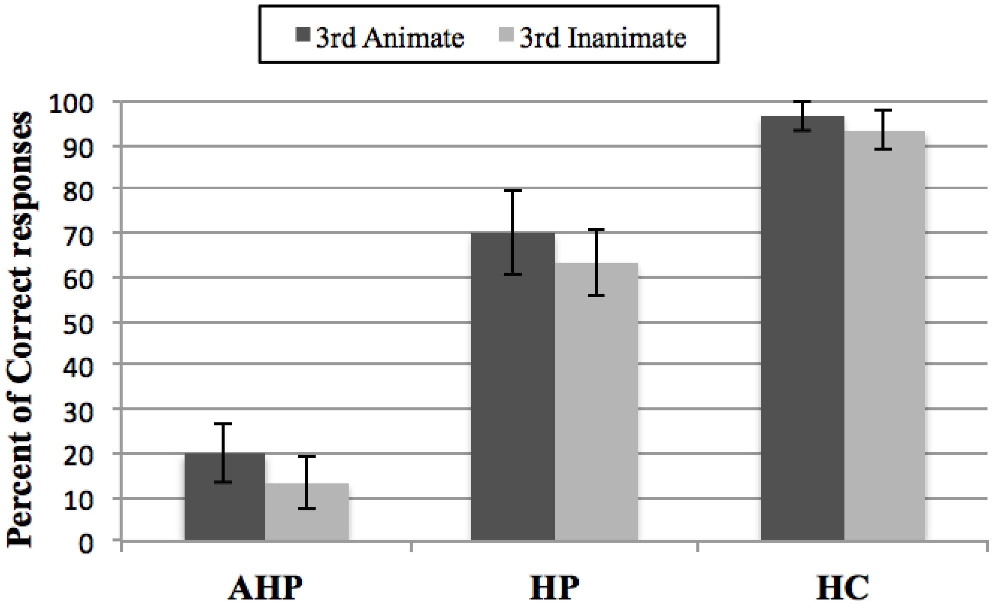

Supplementary Figure 1. Percentage of correct responses across groups (means and SE's) for 3rd animate and 3rd inanimate conditions. There was no significant difference within or between groups.

6. Results of Control tasks

False-belief tasks

In the AHP group, 1 of the 15 patients failed one false belief question in the age-adapted "Smarties" false-belief task, and 4 of the 15 patients failed one false belief question in the Sally-Anne task (93% and 87% of questions passed respectively). All healthy controls, and 14 out of 15 HP passed all false belief questions in both experiments (100% and 97% of questions passed, respectively). The difference between the three groups was not significant for the adapted "Smarties" false-belief task ($H(2)$=1.12, p=0.55) or Sally-Anne task ($H(2)$=3.24, p = 0.2). For both the age-adapted "Smarties" false-belief and Sally-Anne task, all participants passed the reality and memory control questions without exception.

Mental rotation task

A Mann-Whitney-*U* test was used to compare performance on the mental rotation task between the two patient groups (the task was not administered in the healthy control group). The test revealed that there was no significant difference between groups (Z = 0.64, p = 1, r = 0.18; AHP: median = 50, SD = 10.2; HP: median = 50, SD = 12.9).
