# Supplementary material for: Pharmacological targeting of CSF1R inhibits microglial proliferation and prevents the progression of Alzheimer’s-like pathology
Source: Brain. 2016 Jan 8;139(3):891–907. doi: 10.1093/brain/awv379 (PMC4766375; doi:10.1093/brain/awv379)
Supplement: Supplementary Table 1 [file suppl_data.zip › brain-2015-00799-File010.pdf]

Supplementary Table 1. *Comparison of anosognosic and hemiplegic control patients with healthy controls on the visual spatial perspective taking task (using Crawford, Garthwaite & Porter (2010) Revised Standardised Difference Test).*

	1st person perspective[a]			3rd person perspective[b]			Dissociation Test[c]	
**Patient**	**Score (% correct)**	***t***	***p* (1-tailed)**	**Score (% correct)**	***t***	***p* (1-tailed)**	***t***	***p* (1-tailed)**
AHP	100	0	.50	25	-6.55	<.001*	6.12	<.001**
1/RK	100	0	.50	25	-6.55	<.001*	6.12	<.001**
2/GU	100	0	.50	50	-4.35	<.001*	4.04	.001**
3/CA	100	0	.50	0	-8.89	<.001*	8.04	<.001**
4/AB	50	-484	<.001*	25	-6.55	<.001*	302.10	<.001**
5/GA	100	0	.50	25	-6.55	<.001*	6.12	<.001**
6/JT	100	0	.50	50	-4.35	<.001*	4.04	.001**
7/JM	100	0	.50	0	-8.89	<.001*	8.04	<.001**
8/CD	50	-484	<.001*	0	-8.89	<.001*	302.02	<.001**
9/OL	100	0	.50	0	-8.89	<.001*	8.04	<.001**
10/MM	100	0	.50	0	-8.89	<.001*	8.04	<.001**
11/MO	100	0	.50	0	-8.89	<.001*	8.04	<.001**
12/CP	100	0	.50	0	-8.89	<.001*	8.04	<.001**
13/GK	100	0	.50	50	-4.35	<.001*	4.04	.001**
14/SA	100	0	.50	0	-8.89	<.001*	8.04	<.001**
15/IB	100	0	.50	25	-6.55	<.001*	6.12	<.001**
HP Controls								
1/RS	50	-3.50	.002*	50	-4.21	<.001*	302.17	<.001**
2/BD	100	0	.50	50	-4.21	<.001*	4.04	.001**
3/NBD	100	0	.50	0	-8.89	<.001*	8.04	<.001**
4/DS	100	0	.50	100	0.47	.324	0.46	.327
5/BSG	100	0	.50	50	-4.21	<.001*	4.04	.001**
6/ND	100	0	.50	75	-1.87	.041*	1.83	.045**
7/SC	100	0	.50	25	-6.55	<.001*	6.12	<.001**
8/DM	100	0	.50	100	0.47	.324	0.46	.327
9/AM	100	0	.50	75	-1.87	.041*	1.83	.045**
10/FB	100	0	.50	50	-4.21	<.001*	4.04	.001**
11/PR	100	0	.50	75	-1.87	.041*	1.83	.045**
12/IJ	100	0	.50	100	0.47	.324	0.46	.327
13/SM	100	0	.50	100	0.47	.324	0.46	.327
14/KR	100	0	.50	50	-4.21	<.001*	4.04	.001**
15/JR	100	0	.50	100	0.47	.324	0.46	.327

[a] Healthy Control mean = 100; SD = 0 (NB: a value of 0.01 was used in the specialized software to allow calculation of required statistics); N = 15.
[b] Healthy Control mean = 95; SD = 10.53; N = 15.
[c] Correlation between 1st and 3rd person tasks in healthy control sample = .535 (NB: calculation adjusted for ceiling effects as noted for standard deviation).
* significant deficit
** significant dissociation (differential deficit) between unimpaired first person perspective taking ability and third person perspective taking deficit.
